# Dynamics of the Gut Bacteriome During a Laboratory Adaptation Process of the Mediterranean Fruit Fly, *Ceratitis capitata*

**DOI:** 10.3389/fmicb.2022.919760

**Published:** 2022-07-01

**Authors:** Naima Bel Mokhtar, Marta Catalá-Oltra, Panagiota Stathopoulou, Elias Asimakis, Imane Remmal, Nikolaos Remmas, Amal Maurady, Mohammed Reda Britel, Jaime García de Oteyza, George Tsiamis, Óscar Dembilio

**Affiliations:** ^1^Laboratory of Systems Microbiology and Applied Genomics, Department of Environmental Engineering, University of Patras, Agrinio, Greece; ^2^Laboratory of Innovative Technology, National School of Applied Sciences of Tangier, Abdelmalek Essâadi University, Tétouan, Morocco; ^3^Empresa de Transformación Agraria S.A., S.M.E., M.P. (TRAGSA), Paterna, Spain; ^4^Laboratory of Wastewater Management and Treatment Technologies, Department of Environmental Engineering, Democritus University of Thrace, Xanthi, Greece; ^5^Faculty of Sciences and Technology of Tangier, Abdelmalek Essâadi University, Tétouan, Morocco

**Keywords:** medfly, microbial communities, core microbiome, SIT, 16S rRNA, next generation sequencing (NGS)

## Abstract

Laboratory adaptation process used in sterile insect technique (SIT) programs can exert a significant impact on the insect-gut microbiome relationship, which may negatively impact the quality and performance of the fly. In the present study, changes in the gut microbiota that occur through laboratory adaptation of two *Ceratitis capitata* populations were investigated: Vienna 8 genetic sexing strain (GSS), a long-established control line, and a wild population recently introduced to laboratory conditions. The bacterial profiles were studied for both strains using amplicon sequencing of the 16S rRNA V3-V4 hypervariable region in larvae and in the gastrointestinal tract of teneral (1 day) and adults (5 and 15 days) reared under laboratory conditions for 14 generations (F0–F13). Findings demonstrated the development of distinct bacterial communities across the generations with differences in the bacterial composition, suggesting a strong impact of laboratory adaptation on the fly bacteriome. Moreover, different bacterial profiles were observed between wild and Vienna 8 FD-GSS displaying different patterns between the developmental stages. Proteobacteria, mainly members of the *Enterobacteriaceae* family, represented the major component of the bacterial community followed by Firmicutes (mainly in Vienna 8 FD-GSS adults) and Chlamydiae. The distribution of these communities is dynamic across the generations and seems to be strain- and age-specific. In the Vienna 8 FD-GSS population, *Providencia* exhibited high relative abundance in the first three generations and decreased significantly later, while *Klebsiella* was relatively stable. In the wild population, *Klebsiella* was dominant across most of the generations, indicating that the wild population was more resistant to artificial rearing conditions compared with the Vienna 8 FD-GSS colony. Analysis of the core bacteriome revealed the presence of nine shared taxa between most of the examined medfly samples including *Klebsiella, Providencia, Pantoea*, and *Pseudomonas*. In addition, the operational taxonomic unit co-occurrence and mutual exclusion networks of the wild population indicated that most of the interactions were classified as co-presence, while in the Vienna 8 FD-GSS population, the number of mutual exclusions and co-presence interactions was equally distributed. Obtained results provided a thorough study of the dynamics of gut-associated bacteria during the laboratory adaptation of different *Ceratitis capitata* populations, serving as guidance for the design of colonization protocols, improving the effectiveness of artificial rearing and the SIT application.

## Introduction

Colonization of insects under artificial rearing conditions is an important process in several situations, including bioassays, physiological research, postharvest treatment testing, and in large-scale factory production for the release in pest management programs, such as the sterile insect technique (SIT) (Dyck et al., 2021). SIT is a species-specific, sustainable, environmentally friendly approach that involves mass-rearing of the target insects. It relies on the release of sterile male flies aimed to copulate with wild females, causing infertile crosses and subsequent population suppression (Dyck et al., 2021). In most of cases, the SIT approach sterility is induced through irradiation (Knipling, 1959). The successful implementation of a wide area of SIT program requires (1) the ability to rear, sterilize, and distribute enough number of insects at a sustainable cost, and (2) high biological quality sterile males showing high performance and mating ability as that of their wild counterparts (Dyck et al., 2021). However, the colonization and production methods that may lead to the production of flies with improved fitness, are indeed very challenging. The laboratory adaptation processes of the strains used under artificial mass rearing have been reported to improve development, survival, and reproduction under a laboratory environment (Liedo et al., 2007; Diamantidis et al., 2011; Majumder et al., 2022), but at the same time, they commonly have detrimental effects on field performance (Liedo et al., 2007; Pereira et al., 2007; Hernández et al., 2009). The culturing of tephritid fruit flies is generally associated with fast development of flies, increase in body size, and survival while some incompatibility with wild flies may evolve in mating behavior and reduced environmental tolerance (Mangan, 1997; Miyatake, 1998; Briceño and Eberhard, 2002; Zygouridis et al., 2014; Schutze et al., 2015; Pérez et al., 2018).

The symbiotic communities, especially gut microbiota, have been shown to affect different aspects of insect physiology and life history traits (Ben-Yosef et al., 2008; Douglas, 2015; Gupta and Nair, 2020; Ravigné et al., 2022). In tephritid fruit flies, several studies have shown that certain bacterial strains can enhance the productivity of the colonies, such as increasing the female fecundity, pupation rate, and longevity (Behar et al., 2008c; Hamden et al., 2013; Sacchetti et al., 2014; Augustinos et al., 2015) as well as the biological quality of released sterile males, such as flight ability, mating competitiveness, insecticide resistance, and overcoming plant defenses (Ben Ami et al., 2010; Ben-Yosef et al., 2015a; Cheng et al., 2017; Kyritsis et al., 2017; Shuttleworth et al., 2019). Limited number of studies investigated the core microbiome shared between the samples of most of fruit flies. The family, *Enterobacteriaceae* has been reported as the main component of the core microbiome of wild larvae of various tephritid flies (De Cock et al., 2020) and wild adults of *Zeugodacus cucurbitae* (Yong et al., 2019). In terms of abundance and despite the variability between studies (including strain, geographical locations, developmental stage, and rearing conditions), members of the family, *Enterobacteriaceae* have been reported to represent the major component of laboratory and wild tephritid populations, with *Klebsiella, Citrobacter, Enterobacter, Providencia, Proteus*, and *Bacillus* repeatedly detected in different species of fruit flies, including *C. capitata* (Behar et al., 2008c; Malacrinò, 2018; Nikolouli et al., 2020), *Bactrocera dorsalis* (Yong et al., 2017; Zhao et al., 2018), *Bactrocera tryoni* (Woruba et al., 2019; Majumder et al., 2022), *Bactrocera oleae* (Ben-Yosef et al., 2015b; Bigiotti et al., 2021), *Zeugodacus cucurbitae* (Hadapad et al., 2016; Asimakis et al., 2019; Yong et al., 2019), and *Anastrepha fraterculus* (Augustinos et al., 2019; Salgueiro et al., 2020). Moreover, some tephritid fruit flies also harbor *Pseudomonas* (Behar et al., 2008c), *Morganella* (Salas et al., 2017), and *Serratia* (Fitt and O'Brien, 1985) which are often harmless but can be occasional pathogens.

The Mediterranean fruit fly (Medfly), *Ceratitis capitata* (Diptera: Tephritidae), is a serious threat to several agricultural crops worldwide, as it is a highly polyphagous species that is able to attack diverse plant species, and adapt to a wide range of climates (Liquido et al., 1990, 1991; Papadopoulos et al., 2001; Malacrida et al., 2006). Initially, adult females oviposit their eggs under the surface of the fruit. After the hatching of the eggs, the resulting larvae feed on the fruit pulp, reducing both crop yield and the value of the product. In addition, the oviposition holes and the larval tunnel inside the fruit provide entry points for secondary fungal and bacterial infections (Papadopoulos, 2008). The economic losses due to *C. capitata* are amounting to several billions of EUR annually (Szyniszewska and Tatem, 2014). In Spain, *C. capitata* is considered a key pest of economic importance, as it is highly distributed over the country and due to its ability to attack a variety of fruit crops, with citrus fruits being the crop most affected by its populations (Escudero-Colomar et al., 2008; Peñarrubia-María et al., 2012; Juan-Blasco et al., 2014; Tormos et al., 2018). In the Valencian community (Spain), *C. capitata* negatively affects more than 1 million tons of citrus and other fruits (Plá et al., 2021). To overcome this situation, SIT, as a part of area-wide integrated pest management (AW-IPM) strategies, has been successfully used as a control management technique to contain, suppress, or eradicate outbreaks of target populations of *C. capitata* in numerous countries including Spain (Enkerlin and Mumford, 1997; Hendrichs et al., 2002; Calkins and Parker, 2005; Suckling et al., 2016; Plá et al., 2021).

Genetic sexing strains (GSSs) have been developed for *C. capitata* (such as the Vienna 7 and Vienna 8 GSSs) (Augustinos et al., 2017); they are widely used in mass rearing facilities to enhance SIT applications because they allow for the large-scale release of only sterile males in the field (females are temperature-sensitive and could be killed through heat treatment during the last stage of the embryonic development), thus reducing the overall cost and avoiding the negative impact related to the release of females (Hendrichs et al., 1995; Cáceres et al., 2000; Cáceres, 2002; Franz, 2005). The Vienna 8 fast development strain (Vienna 8D53-FD GSS) is a new GSS originating from the Vienna 8 temperature-sensitive lethal GSS that was developed by FAO/IAEA at the Insect Pest Control Laboratory (IPCL) in Seibersdorf, Austria. This novel strain, which combines the temperature sensitive lethal (tsl) and the white pupae (wp) genes as selectable markers, shows faster development in females during the larval stage and increased temperature sensitivity compared with the regular Vienna 8 strain (Porras et al., 2020). The introgression of this novel wp tsl FD combined trait into the Vienna 8D53 GSS, resulted in a novel Vienna 8D53-FD GSS, where females showed differences in the thermal sensibility, larval development speed, and productivity profiles (Porras et al., 2020). Currently, it is under small-scale laboratory tests and could be a potential candidate to be implemented in mass-rearing conditions in the SIT program in the Valencian Community.

To ensure the efficacy of SIT applications and given the importance of the gut bacteria on insect fitness, it is important to elucidate how the bacterial structure and diversity change during the laboratory adaptation of *C. capitata* colonies. Thus, in this study, using high-throughput Illumina sequencing of 16S rRNA gene, the gut bacterial communities of *C. capitata* reared across 13 generations under artificial conditions were characterized. Colonies were established from two *C. capitata* strains (1) wild type collected from citrus orchards in Valencia and (2) novel Vienna 8D53-FD GSS. To examine the factors that may contribute to the structuring of gut bacterial communities, at each generation, samples representing different developmental stages, age, and sex, were analyzed. A hypothesis was created that microbial associations hosted by *C. capitata* are modified over the laboratory colonization and vary among the origin strains and developmental stage, while maintaining a shared core microbiome. The effect of diet on larval microbial community in terms of adaptation and major effects on their microbial composition was also assessed.

## Materials and Methods

### Medfly Colonies, Origins, and Maintenance

The experiment was conducted using two medfly strains: one stablished from wild individuals reared under laboratory conditions, and the second one with the Fast Development strain (Vienna 8D53-FD GSS, hereafter referred to as Vienna 8 FD-GSS) (Porras et al., 2020). The wild-type colony came from field-collected *C. capitata* pupae obtained from infested mandarin orange (*Citrus reticulada* and *C. unshiu*) collected from an experimental citrus orchard located in Instituto Valenciano de Investigaciones Agrarias (IVIA) (Valencia, Spain) in October of 2018. Larvae from these infested fruits (F0) were allowed to jump and pupate in wheat bran. Once the emergence of adults took place, they were transferred to an expanded polystyrene cage with two mesh windows (30 x 40 x 8 cm) and fed with a mixture of sugar and hydrolyzed yeast extract (3:1, w:w) and water. Mangoes were offered to females through one of the mesh windows for egg laying. Immature stages (larvae) were developed inside the mangoes until they jumped into wheat bran, and the pupae were collected. Larvae from F1 to F8 were developed in the same host fruit (mangoes). After these generations, females were capable to lay eggs through the mesh without fruit; these eggs fall into a tray containing water where they were collected and transferred to an artificial diet for larval development (652.3 ml of water, 115 g of sugar, 101.7 g of brewer's yeast, 3.10 g of benzoic acid, 3.70 g of methyl paraben, and 6 ml of 35% HCl to prepare 1 kg of diet; pH adjusted to 3.8 ± 0.1 with HCl 35%). Adults of all generations were maintained as described above for F0 adults. This colony was daily maintained at the facilities of the IVIA by state-owned company Empresa de Transformación Agraria (TRAGSA), S.A., S.M.E., M.P., Spain, and the environmental conditions were the same for larvae and adults throughout the whole experiment, 25 ± 3°C, 60–65% relative humidity with a photoperiod of 14:10 (L: D) (Supplementary Table 1).

The new Vienna 8 FD-GSS used in the experiment were kindly provided by FAO/IAEA Seibersdorf laboratory in February 2019 (F56 generation) and were reared following the standard procedures established in our laboratory. Larvae and pupae of this strain were delivered to the mass-rearing facility located in Caudete de las Fuentes (Valencia, Spain) (larvae preserved in 70% of ethanol) and this rearing was considered, at that point, as F0 under our conditions. Emerged F0 of Vienna 8 FD-GSS adults were transferred to an expanded polystyrene cage (30 x 40 x 8 cm) with a mesh window and fed with a mixture of sugar and hydrolyzed yeast extract (3:1, w:w) and water. Female laid eggs through the mesh which fell into a tray containing water; they were collected and transferred to the artificial diet (the same as described above). Adults and immature stages of all generations were maintained following the same procedure. The environmental conditions were 26 ± 2°C, 65–70% relative humidity in darkness for larvae/pupae and 24 ± 2°C, 60–65% relative humidity with a photoperiod of 14:10 (L:D) for adults.

### Preparation of Medfly Samples

Larvae, teneral, and adults of both strains used in this study were obtained from the F0, F1, F2, F4, F7, F9, F11, and F13 generations. For each indicated generation, five individuals of mature third instar larvae were collected and then rinsed four times individually with 0.01 of sterile phosphate-buffered saline (PBS) and preserved in 1.5 ml microtubes containing 750 μl of 100% ethanol at−20°C (five gut larvae per replicate). F0 wild type larvae were extracted directly from infested mandarins collected from the field. For adults, guts were collected from 1 day old (teneral), 5 and 15 days old (adults) for both sexes, males, and females separately. A pool of teneral and adult flies was sampled from each colony and from each generation for evaluation purposes and held until gut extraction in methacrylate cages (30 x 30 x 30 cm) with a mesh window and fed with a mixture of sugar and hydrolyzed yeast extract (3:1, w:w) and water. Teneral flies were only water supplied. Cages were kept inside an environmental chamber at 25 ± 2°C, 60–70% relative humidity with a photoperiod of 14:10 (L:D). Teneral and adults were aseptically dissected to extract the guts under a binocular microscope, using sterile tweezers and micropins. While the extractions were being performed, 0.01 M of a sterile PBS was used to prevent gut desiccation during the removal of non-target body tissues and maintain their integrity (midguts, malpighian tubes, and hindgut). The guts were then individually rinsed 4 times with fresh PBS solution and a pool of five guts was prepared per sample and maintained in 750 μl of 100% ethanol as larvae samples. In total, 324 samples (including three biological replicates for each sample) were analyzed (Supplementary Table 1).

### DNA Isolation, Library Preparation, and Illumina MiSeq Sequencing

Total DNA extraction was performed following a modified CTAB protocol (Doyle and Doyle, 1987). The quality of DNA preparations and the concentration of double-stranded DNA were estimated using a Q5000 micro-volume UV Vis spectrophotometer (Quawell Technology, San Jose, CA, USA).

Polymerase chain reaction was performed using KAPA Taq Polymerase kit (KAPA BioSystems). The variable V3–V4 region of the bacterial 16S rRNA sequences was amplified using fusion primers U341F-MiSeq and 805R-MiSeq (Klindworth et al., 2013). Each 25 μl reaction contained KAPA Taq Buffer (10X) at a final concentration of 1X, dNTP mix solution at 200 μM each, forward and reverse primer solution at 0.4 μM, 0.5 U of KAPA Taq DNA polymerase (5 U/μl), ≤ 250 ng from the template DNA solution, and sterile deionized water. The amplification protocol included a 3 min incubation at 95°C followed by 35 cycles of 95°C for 30 s, 55°C for 30 s and 72°C for 1 min, and a final 1 min extension at 72°C. Negative and positive controls were always performed in parallel. PCR products were separated in a 1.5 % (w/v) of agarose gel in TAE buffer (1X) (40 mM Tris–acetate, 1 mM EDTA). Approximately, 550 bp amplification products were visualized in Bio-Rad's Gel Doc™ XR+ system. Positive PCR fragments were then purified from primers and primer dimers by mixing the PCR product with an equal volume of PEG solution (20 % PEG 8000, 2.5 M NaCl), incubating at 37°C for 15 min, centrifuging at 14,000 x g for 20 min, and washing the precipitate twice with 125 μl of 70% v/v ethanol solution and centrifuging at 14,000 x g for 10 min. The dried precipitates were suspended in 15 μl of sterile deionized water and their concentration was measured with a Quawell Q5000 micro-volume UV-Vis spectrophotometer.

Purified PCR amplicons were diluted up to 10 ng/μl and used as templates within the second-step PCR to include the Illumina barcodes. The combinatorial use of index primers resulted in unique samples that were pooled and sequenced using an Illumina MiSeq. In more detail, amplification was performed using the KAPA Taq Polymerase (KAPA BioSystems, USA) in a final volume of 50 μl. Each reaction contained 5 μl of KAPA Taq Buffer (10X), 0.6 μl of dNTPs solution (25 mM each), 5 μl of the forward indexing primer (10 μM), 5 μl of the reverse indexing primer (10 μl), 0.4 μl of KAPA Taq DNA Polymerase (5 U/μl), and 2 μl from the diluted PCR product (10 ng/μl) and sterile deionized water. The PCR amplifications were performed with a 3 min incubation at 95°C followed by eight cycles of 95°C for 30 s, 55°C for 30 s, and 72°C for 30 s, and a final 5 min terminator reaction at 72°C. The resulting amplicons were purified using NucleoMag^®^ NGS Clean-up and Size Selection kit (Macherey-Nagel, Düren, Germany) according to the manufacturer's recommendations. Amplicons from different samples were quantified with a Quawell Q5000 micro-volume UV-Vis spectrophotometer and merged in equimolar ratios (8 nM). High-throughput sequencing was performed by Macrogen using a 2 × 300 bp pair-end kit on a MiSeq platform. The NCBI Bioproject accession number for the raw sequencing data reported in this study is PRJNA822871.

### Sequence Data Processing and Statistical and Network Analyses

Raw sequencing reads were de-multiplexed and converted to FASTQ, and the Illumina adapters were trimmed using Illumina standard algorithms. The bioinformatic analysis was performed using a combination of USEARCH v.11 (Edgar, 2010) and Qiime2 distribution 2019.1 (Bolyen et al., 2019). Briefly, raw forward and reverse reads of each sample were assembled into paired-end reads, trimmed by length, and merged in a single fastq file using fastq_mergepairs command. The paired-end reads were quality filtered and the duplicated sequences were removed using fastq_filter and fastx_uniques commands, respectively. Thereafter, assembled reads were clustered into operational taxonomic units (OTUs) at 97% sequence similarities using cluster_otus command based on UPARSE algorithm (Edgar, 2013). Cross-talk errors were identified and filtered with uncross command based on UNCROSS2 algorithm (Edgar, 2018). The extremely rare OTUs (<0.001% of total sequences across all samples) were discarded using otutab_trim command. Taxonomy was assigned to the representative sequences of the OTUs in Qiime2 based on BLAST+ algorithm (Camacho et al., 2009) and searching against SILVA 128 release database (Quast et al., 2013) with a 0.91% identity as cutoff. Phylogenetic tree was constructed using FastTree (Price et al., 2009); then the phylogeny was rooted using midpoint-root method as implemented in Qiime2. The OTUs table, the taxonomy table, and the rooted phylogenetic tree were used as inputs for the subsequent analyses.

Alpha diversity indices, as well as indices depicting the population structure (observed OTUs and goods coverage) were calculated based on a normalized OTU table at a depth of 5,000 sequences/sample. Richness, Simpson, Shannon, and Evenness indices of alpha diversity, which reflect the diversity of individual samples were calculated based on vegan R package (Oksanen et al., 2020) and plotted using ggplot2 R package (Wickham, 2016). Pairwise ANOVA was used to identify significant differences of alpha diversity indices between the different groups.

Core bacterial taxa detected in 75% of the samples with a relative abundance threshold value above 0.01%, were identified using core function in microbiome R package (Lahti and Shetty, 2019) based on the whole *C. capitata* dataset. In order to investigate the conserved, recruited, and excluded taxa during the laboratory adaptation, the core bacterial taxa with at least 75% prevalence were determined in each generation of the laboratory adaptation for both Vienna 8 FD-GSS and wild population. Core bacterial taxa were then compared between developmental stages of both strains.

Between samples diversity was calculated based on Generalized UniFrac distance (Chen et al., 2012) using GUniFrac R package (Chen et al., 2021). Canonical analysis of principal coordinates (CAPs) (Anderson and Willis, 2003) based on 999 permutation tests, principal coordinates analysis (PCoA) and non-metric multidimensional scaling (NMDS) were performed on the resulting dissimilarity matrix. Statistically significant differences between samples were identified with permutational multivariate analysis of variance (PERMANOVA) (Anderson, 2001) using 999 permutations and Monte Carlo tests. CAP, PCoA analyses and PERMANOVA test were performed on PRIMER version 6 and PERMANOVA+ for PRIMER routines (Clarke and Gorley, 2006; Anderson et al., 2008). NMDS ordination was visualized using ggpubr R package (Kassambara, 2020). Pairwise comparisons of mean relative abundance of OTUs between gut samples were performed using non-parametric Wilcoxon rank sum test (Bauer, 1972). The obtained significance values were corrected for multiple testing using the Benjamini-Hochberg method (Benjamini and Hochberg, 1995).

Interactions between microorganisms were investigated and visualized through co-occurrence networks. These interactions refer to microorganisms performing similar or complementary functions and/or sharing similar environmental conditions, but not necessarily having physical interactions (Steele et al., 2011; Zhou et al., 2011). Network analysis was performed independently based on significant grouping of generations identified by beta diversity. Co-presence and mutual exclusion interactions among OTUs selected in each generation (group of generations) were identified using CoNet plugin (Faust and Raes, 2016) in Cytoscape (Shannon et al., 2003) using the following ensemble of methods: Pearson and Spearman correlation coefficients, and the Bray Curtis and Kullback–Leibler dissimilarity indices. Statistical significance of each interaction was tested using the row-shuffle randomization followed by bootstrap score distributions options with 1,000 iterations. Edges with original scores outside the 0.95 range of their bootstrap distribution were discarded, and the merged *p*-values were corrected using the Benjamini–Hochberg method (Benjamini and Hochberg, 1995).

## Results

### Analysis of Bacterial 16S rRNA Sequences

Bacterial community composition and diversity of *C. capitata* populations were investigated by sequencing the V3-V4 region of the 16S rRNA gene producing a total of 13,799,084 reads. After filtering low quality sequences, 10,273,916 reads were clustered into OTUs at 97% identity threshold with an average of 31,709 reads/sample. After grouping the three replicates, the number of reads per sample ranged from 6,282 to 66,794, providing a high coverage (>99%) of the existing bacterial diversity based on the good's coverage index (Supplementary Table 2). A set of 52 distinct OTUs were present at a relative abundance of over 0.1% within the dataset and were classified in three phyla, four classes, 16 families, and 40 genera (Supplementary Table 3). At the phylum level, Proteobacteria was the most abundant taxonomic group, followed by Firmicutes and minor Chlamydiae communities. Within Proteobacteria, most of the bacterial sequences found in *C. capitata* belong to the family, *Enterobacteriaceae* representing 69.9% of the total bacterial sequences followed by *Halomonadaceae* (5.45%) and members of the Firmicutes phylum, such as *Streptococcaceae* (5.1%) and *Enterococcaceae* (4.7%). At genus level, *Klebsiella* was the most abundant taxon (25.9%) followed by *Providencia* (15.3%), *Pluralibacter* (9.3%), *Pantoea* (5.5%), and *Carnimonas* (5.4%).

### Core Microbiome During the Laboratory Colonization Process

**Figure 1:**
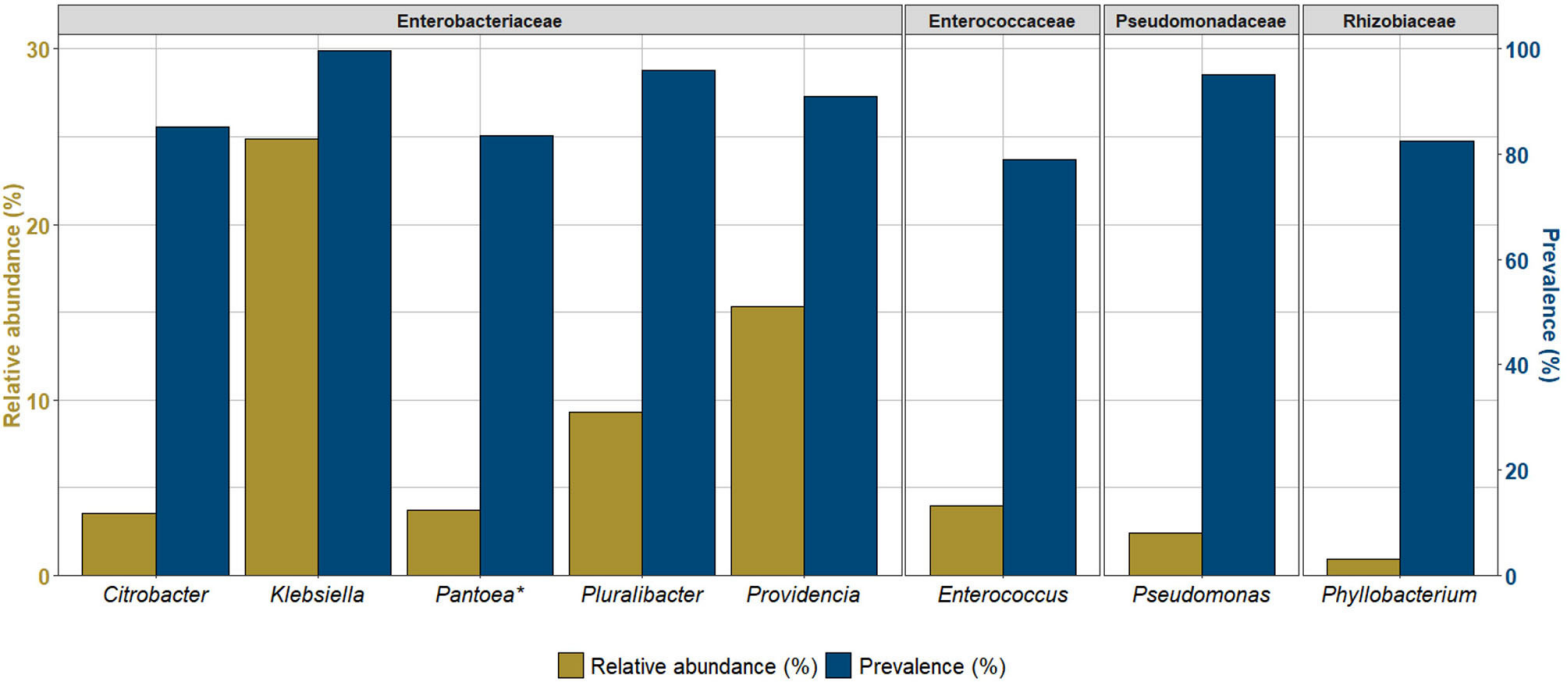
Overall core bacterial taxa identified in at least 75% of the samples of *Ceratitis capitata*. Asterisk (^*^) represents genera identified in as two variants.

A total of nine OTUs were identified as the core bacteriome of *C. capitata* at a 75% prevalence threshold representing together ~65% of the total bacterial community. Its primary component included members of the *Enterobacteriaceae* family (six OTUs) followed by *Pseudomonadaceae, Rhizobiaceae* and *Enterococcaceae*. *Klebsiella* displayed a prevalence in 99% of the samples, *Pluralibacter* and *Pseudomonas* a prevalence of 95%, *Providencia* in 91% and *Citrobacter, Pantoea* (2 variants), *Phyllobacterium* and *Enterococcus* were found in 79–85% of the samples (Figure 1, Supplementary Table 4).

Core genera associated to Vienna 8 FD-GSS samples are very similar to the wild population samples examined during the laboratory adaptation event. *Klebsiella* and *Providencia* were identified in the three developmental stages examined over all the generations except for F9 and F11, where *Providencia* was below detection levels in adult flies (**Table 2**, Supplementary Table 5). *Pseudomonas* was identified mainly in teneral and adult flies over the generations, while at the larval stage, it was detected from the F1 generation onwards (Table 1). Interestingly, *Geobacillus* together with *Enterococcus* were acquired during the F4 generation and established as core bacterial taxa. Although *Carnimonas* displayed a similar behavior, it became part of the core bacterial taxa only in the larval stage. On the other hand, some examined bacterial genera were lost during the laboratory adaptation process with *Morganella* and *Moellerella* being the most prominent examples disappearing after the F4 generation (Table 1, Supplementary Table 5).

**Table 1:** Core bacterial taxa determined at each generation of laboratory adaptation of Vienna 8 FD-GSS *C. capitata* at 75% threshold.

**Taxonomy**					**Larva**								**Teneral**							**Adult**							
**Phylum**	**Class**	**Order**	**Family**	**Genus**	**F0**	**F1**	**F2**	**F4**	**F7**	**F9**	**F11**	**F13**	**F0**	**F1**	**F2**	**F4**	**F7**	**F9**	**F11**	**F0**	**F1**	**F2**	**F4**	**F7**	**F9**	**F11**	**F13**
Proteobacteria	Gammaproteobacteria	Enterobacteriales	Enterobacteriaceae	Klebsiella	P	P	P	P	P	P	P	P	P	P	P	P	P	P	P	P	P	P	P	P	P	P*	P
Proteobacteria	Gammaproteobacteria	Enterobacteriales	Enterobacteriaceae	Moellerella									P	P	P					P	P	P	P				
Proteobacteria	Gammaproteobacteria	Enterobacteriales	Enterobacteriaceae	Morganella	P	P	P	P					P	P						P	P	P					
Proteobacteria	Gammaproteobacteria	Enterobacteriales	Enterobacteriaceae	Providencia	P	P	P	P	P	P	P	P	P	P	P	P	P	P	P	P	P	P	P	P			P
Proteobacteria	Gammaproteobacteria	Oceanospirillales	Halomonadaceae	Carnimonas				P	P	P	P	P															
Proteobacteria	Gammaproteobacteria	Pseudomonadales	Pseudomonadaceae	Pseudomonas		P	P	P		P	P	P	P	P	P	P	P	P	P	P	P	P	P	P	P		P
Firmicutes	Bacilli	Bacillales	Bacillaceae	Geobacillus						P	P	P				P	P	P	P				P		P		P
Firmicutes	Bacilli	Lactobacillales	Enterococcaceae	Enterococcus					P	P	P	P	P	P		P	P*		P	P*	P	P	P*	P*	P	P	P

“P” (Present) represents the identification of taxon at each generation. Asterisk (^*^) represents genera identified as two variants.

The core microbiome during the laboratory adaptation process of the wild *C. capitata* population was comparable to Vienna 8 FD-GSS-associated core microbiome with regard to the composition as well as the shared OTUs across generations. Different bacterial genera with different relative abundance levels were conserved over the laboratory adaptation of larva, teneral, and adults, including *Klebsiella, Pseudomonas*, and *Pluralibacter*. *Pantoea* (two variants) and *Citrobacter* were conserved over all generations examined for the teneral and adult flies and only at the larval stage could not be detected at F9 and F11 generation, respectively (Table 2, Supplementary Table 6). *Providencia* was highly conserved at the larval stage; however, at the teneral and adult stages, it was not present at F13 and F9 generations, respectively (Table 2). Similarly, to Vienna 8-GSS, *Geobacillus* displayed an analogous behavior being acquired during the F4 generation and established as a core bacterial taxon. While *Carnimonas* joined the core bacterial taxa of larva in the last generations starting from F11. Conversely, in the last generations of the laboratory adaptation process, *Morganella* was lost from the core microbiome of the three studied developmental stages (from F9 for larva and adults and F13 for teneral). Meanwhile, *Acetobacter* was exclusively identified in wild larva before laboratory adaptation (F0) representing 83.3% of the bacterial community (Table 2, Supplementary Table 6).

**Table 2:** Core bacterial taxa determined at each generation of laboratory adaptation of wild *C. capitata* at 75% threshold.

**Taxonomy**					**Larva**								**Teneral**								**Adult**							
**Phylum**	**Class**	**Order**	**Family**	**Genus**	**F0**	**F1**	**F2**	**F4**	**F7**	**F9**	**F11**	**F13**	**F0**	**F1**	**F2**	**F4**	**F7**	**F9**	**F11**	**F13**	**F0**	**F1**	**F2**	**F4**	**F7**	**F9**	**F11**	**F13**
Proteobacteria	Gammaproteobacteria	Enterobacteriales	Enterobacteriaceae	Citrobacter	P	P	P	P	P	P	P		P	P	P	P	P	P	P	P	P	P	P	P	P	P	P	P
Proteobacteria	Gammaproteobacteria	Enterobacteriales	Enterobacteriaceae	Klebsiella	P	P	P	P	P	P	P	P	P	P	P	P	P	P*	P	P	P	P	P	P	P	P*	P	P
Proteobacteria	Gammaproteobacteria	Enterobacteriales	Enterobacteriaceae	Morganella	P	P	P	P	P				P	P	P	P		P	P		P	P	P	P	P			
Proteobacteria	Gammaproteobacteria	Enterobacteriales	Enterobacteriaceae	Pantoea	P*	P*	P*	P	P*	P*			P*	P*	P*	P*	P*	P*	P*	P	P*	P*	P*	P*	P*	P	P*	P*
Proteobacteria	Gammaproteobacteria	Enterobacteriales	Enterobacteriaceae	Pluralibacter	P	P	P		P	P	P	P	P	P	P	P	P	P	P	P	P	P	P	P	P	P	P	P
Proteobacteria	Gammaproteobacteria	Enterobacteriales	Enterobacteriaceae	Providencia	P	P	P	P	P	P	P	P	P	P	P	P	P	P	P		P	P	P	P	P			
Proteobacteria	Gammaproteobacteria	Oceanospirillales	Halomonadaceae	Carnimonas							P	P																
Proteobacteria	Gammaproteobacteria	Pseudomonadales	Pseudomonadaceae	Pseudomonas	P	P	P	P	P	P	P	P	P	P	P	P	P	P	P	P	P	P	P	P	P	P	P	P
Proteobacteria	Alphaproteobacteria	Acetobacterales	Acetobacteraceae	Acetobacter	P																							
Firmicutes	Bacilli	Bacillales	Bacillaceae	Geobacillus				P	P	P	P	P					P	P	P						P			

“P” (Present) represents the identification of taxon at each generation. Asterisk (^*^) represents genera identified as two variants.

### Comparison of Vienna 8 FD-GSS Gut-Associated Bacterial Communities Over Generations at Different Developmental Stages

For the larval stage, CAP, NMDS, and PERMANOVA analyses indicated that the bacterial community changed over the generations (PERMANOVA; *p* < 0.05). Meanwhile, similarities were observed between generations F0 and F1 (PERMANOVA; F0/F1: *p* = 0.14), and F7, F9, F11, and F13 (PERMANOVA; F7/F9/F11/F13: p>0.05) (Supplementary Figures 1A,B). The bacterial community within samples of generation F2 had significantly lower species richness and diversity compared to the other generations while no significant differences in species richness or diversity were observed between the other generations (Supplementary Figure 1C). Overall, the bacterial community associated with *C. capitata* larva for all generations examined was primarily dominated by Proteobacteria (more than 90% of the bacterial community in all generations) followed by Firmicutes and a small Chlamydiae community was observed in F7, F9, F11, and F13 generations (Supplementary Figure 2A). The dominant class of Proteobacteria was Gammaproteobacteria representing 95.7 ± 0.7%, 99.9 ± 0.1%, 80.9 ± 3.2%, and 88.9 ± 2.5% in F0-F1, F2, F4, and F7–F11 groups, respectively (Supplementary Figure 2B). At genus level, the bacterial community composition varied between the first and last generations, where F0–F1 and F2 generations were mainly dominated by *Providencia* (*Enterobacteriaceae*) (66.7 ± 9.6% and 96.7 ± 1.2%, respectively) while F4 and F7–F13 were mainly dominated by *Carnimonas* (*Halomonadaceae*) (55.5 ± 5.5% and 64.9 ± 9.0%, respectively) (Supplementary Figure 2C). In addition, F4 generation was also characterized by a significant increase of *Pseudomonas* (17.8 ± 2.1%; Wilcoxon's rank sum test: *p* < 0.05) (Supplementary Figure 2C).

**Figure 2:**
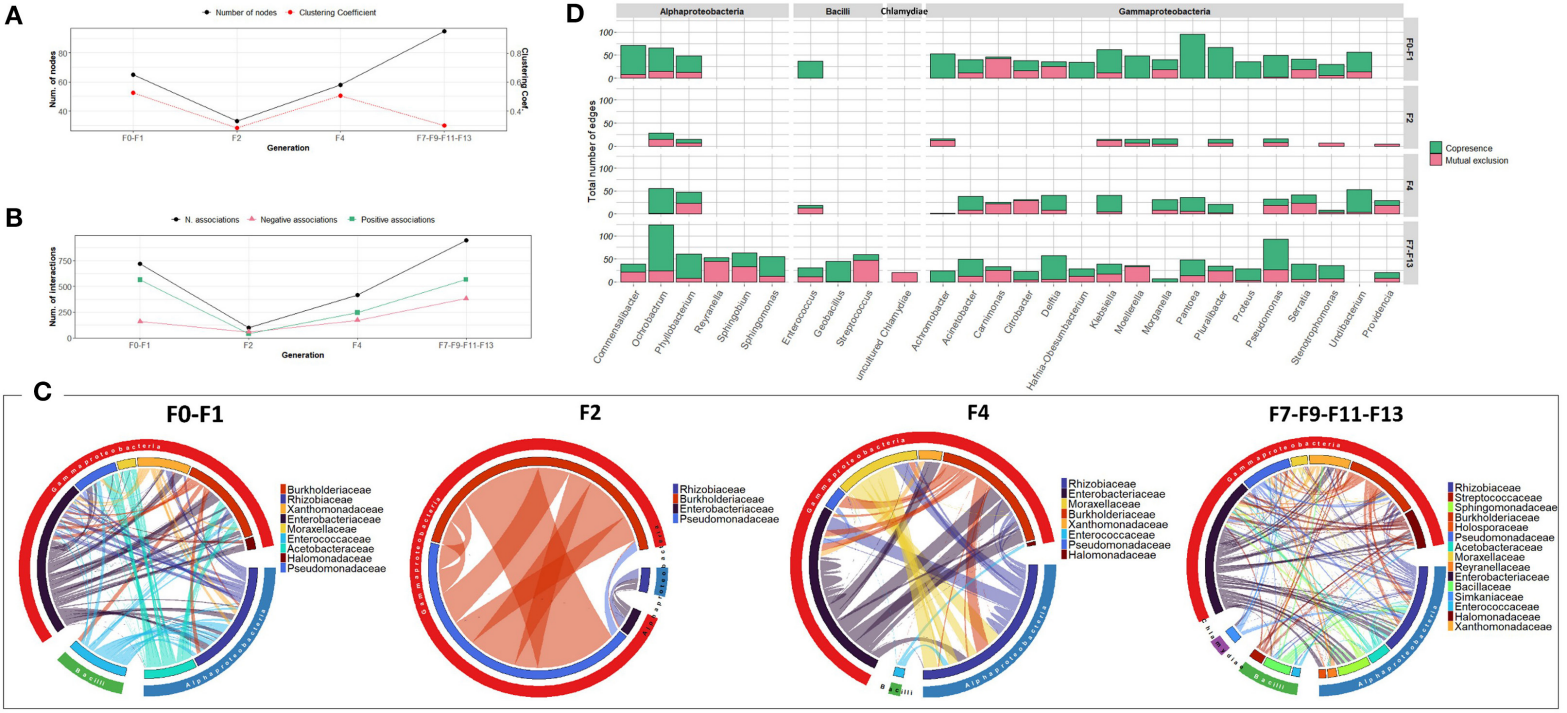
Bacterial community network within the guts of *C. capitata* larva during laboratory adaptation of Vienna 8 FD-GSS strain. **(A)** Total number of nodes and clustering coefficient. **(B)** Total number of negative (red) and positive (green) associations, **(C)** Chord diagram displaying direct relationship between OTUs at family level. The size of the ribbon is proportional to the weight of the association between the OTUs assigned to the respective segments and the color of the ribbon present in the direction of the interaction. **(D)** Number of positive and negative associations at genus level.

The bacterial co-occurrence networks showed marked differences across generations. Overall, a higher number of nodes was observed in the last generations (F7, F9, F11, and F13 groups) which reflects the contribution of different OTUs in the formation of the bacterial community (Figure 2A). The number of associations dropped from 721 to 99 from F0-F1 to F2 generations and then increased in the subsequent generations (417 and 950 in F4 and F7-F9-F11-F13 generations, respectively) indicating a high turnover of OTUs serving as connections with positive associations outnumbering the mutual exclusions (Figure 2B). At F0-F1 generation, members of the *Enterobacteriaceae* family were highly associated with other families of Gammaproteobacteria as well as members of Alphaproteobacteria mainly with *Rhizobiaceae*, while, in the later generations, the associations were shared equally between members of all the contributed families (Figure 2C). Interestingly, despite the dominance of *Providencia* (*Enterobacteriaceae*) at F2, most of the interactions were observed between members of *Burkholderiaceae* and *Pseudomanadaceae* (Figure 2C). At genus level, members of Gammaproteobacteria, such as *Pantoea* and *Pluralibacter* followed by members of Alphaproteobacteria, such as *Commemsalibacter* and *Ochrobacterium*, exhibited the highest degree of co-presence associations at F0–F1 generations. For F7, F9, F11, and F13 generations, various bacterial genera belonging to different classes of Gammaproteobacteria, Alphaproteobacteria, Bacilli, and Chlamydiia have contributed to the bacterial network (Figure 2D).

Teneral flies of Vienna 8 FD-GSS at F0 and F1 generations tend to share similar bacterial profiles (PERMANOVA; F0/F1: *p* = 0.18), while a significant difference was observed between the subsequent generations especially between F4, F7, F9, and F11 which formed different clusters (PERMANOVA; *p* < 0.05; Supplementary Figures 3A,B). A stable species-richness and diversity were observed within F0 to F7 generations with significant reduction of both indices only in the F2 generation. However, higher bacterial diversity was observed for F9 and F11 generations (Supplementary Figure 3C). Proteobacteria was the prevailing phylum across all generations followed by Firmicutes especially from the F4 generation onwards (20.7 ± 5.0%, 8.8 ± 3.0%, 9.3 ± 3.6%, and 15.0 ± 1.3% at F4, F7, F9, and F11, respectively) and minor Chlamydiae communities that were significantly increased in F9 and F11 generations (2.3 ± 0.5% and 6.1 ± 2.2% respectively; Wilcoxon's rank sum test: *p* < 0.05) (Supplementary Figure 4A). Among Proteobacteria, Gammaproteobacteria were the most dominant class across the first generations (F0-F1 and F2) especially in the F2 where they represent 99.9 ± 0.1% of the bacterial community. The decrease of relative abundance of Gammaproteobacteria in the subsequent generations was associated with an increase for members of Bacilli, Alphaproteobacteria, and Chlamydiae (Supplementary Figure 4B). In Gammaproteobacteria, higher relative abundance of the members of the family, *Enterobacteriaceae* was observed, such as *Providencia* in F0–F1 and F2, *Klebsiella* in F4, and *Pluralibacter* in F7. However, F9 and F11 harbor a wide range of bacterial genera including *Pseudomonas* that represent around 17% of the total bacterial communities (Supplementary Figure 4C).

**Figure 3:**
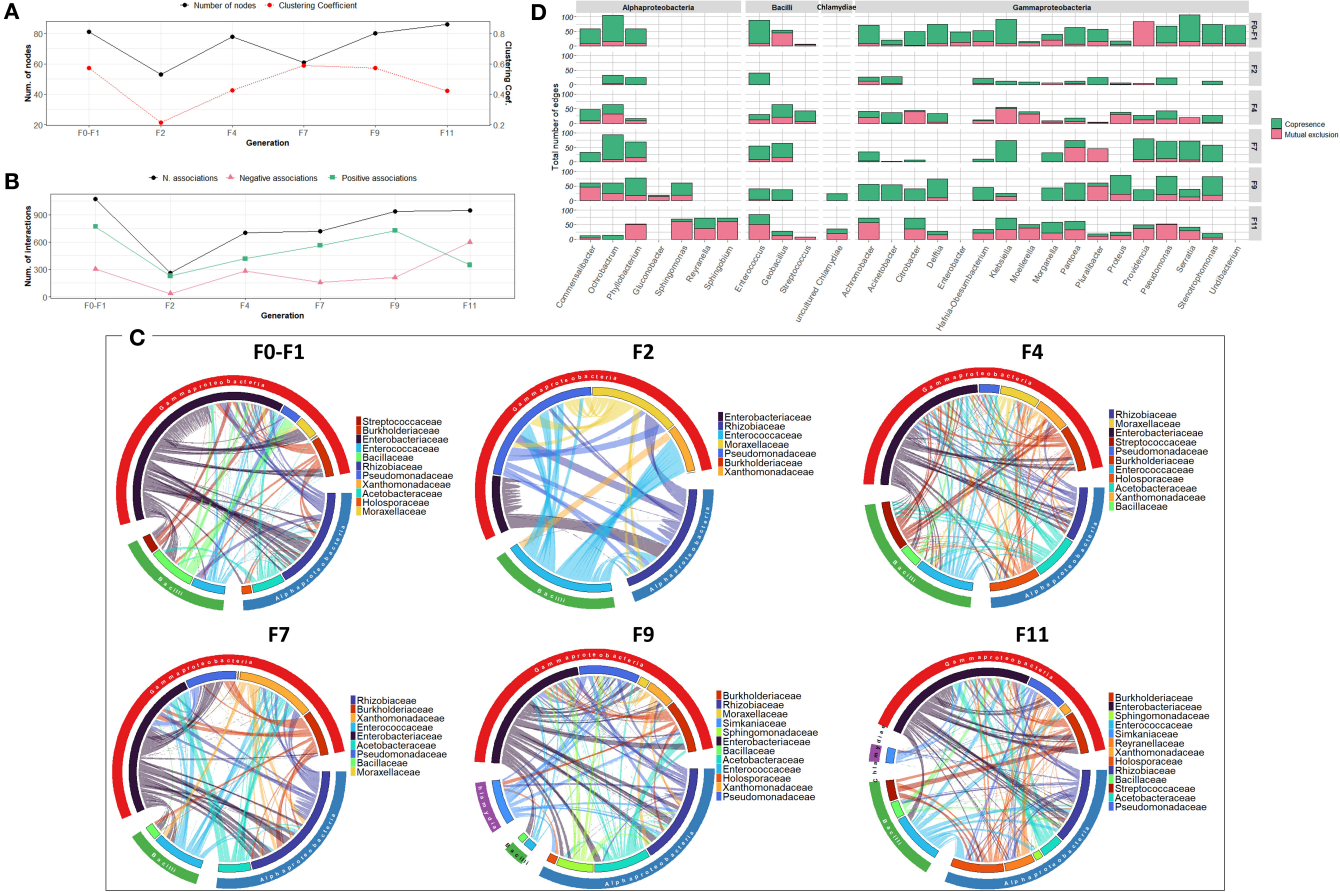
Bacterial community network within the guts of *C. capitata* teneral during laboratory adaptation of Vienna 8 FD-GSS strain. **(A)** Total number of nodes and clustering coefficient. **(B)** Total number of negative (red) and positive (green) associations. **(C)** Chord diagram displaying direct relationship between OTUs at family level. The size of the ribbon is proportional to the weight of the association between the OTUs assigned to the respective segments and the color of the ribbon present in the direction of the interaction. **(D)** Number of positive and negative associations at genus level.

Regarding the bacterial co-occurrence networks, limited variability was observed over the generation in terms of the number of OTUs contributing to the networks and their associations (Figures 3A,B). However, an exception was observed for F2 where a reduced number of interactions was observed which might be due to low bacterial richness and diversity. Across generations, members of *Enterobacteriaceae* were highly associated with other members of Gammaproteobacteria, Alphaproteobacteria and Bacilli, with the exception of F2 generation where they interact only with members of *Rhizobiaceae* (Alphaproteobacteria) (Figure 3C). It is worth noting that the bacterial associations at F11 generation were characterized by an increase in the number of negative associations between different families compared to previous generations (Figure 3D).

Vienna 8 FD-GSS adults also tend to share similar bacterial profiles in the F0 and F1 generations and develop statistically different bacterial profiles over the subsequent generations (PERMANOVA; *p* < 0.05; Supplementary Figures 5A,B). The bacterial community for the last generations F11 and F13 showed higher species-richness and diversity compared to the previous generations (Supplementary Figure 5C). Over the generations, Proteobacteria was the dominant phylum followed by Firmicutes and minor Chlamydiae communities detected only in F7 and F13 (Supplementary Figure 6A). Increasing relative abundance of Bacilli was observed through all the generations examined until reaching its maximum in the F13 generation where it represents 47.6 ± 5.6% of the bacterial community at the expense of members of Gammaproteobacteria. The F2 generation was mainly dominated by members of Gammaproteobacteria (96.8 ± 1.1%) (Supplementary Figure 6B). At family and genus level, fluctuation was observed through generations and more specifically, members of *Enterobacteriaceae* dominated the bacterial communities during the generations from F0 to F9. *Providencia* was dominant in F0-F1 and F2 generations while *Pluralibacter* was dominant in F7 and F9. The F11 and F13 generations, were dominated by *Lactococcus* (*Streptococcaceae*), *Enterococcus* (*Enterococcaceae*), and *Consenzaea* (*Enterobacteriaceae*) (Supplementary Figure 6C).

**Figure 4:**
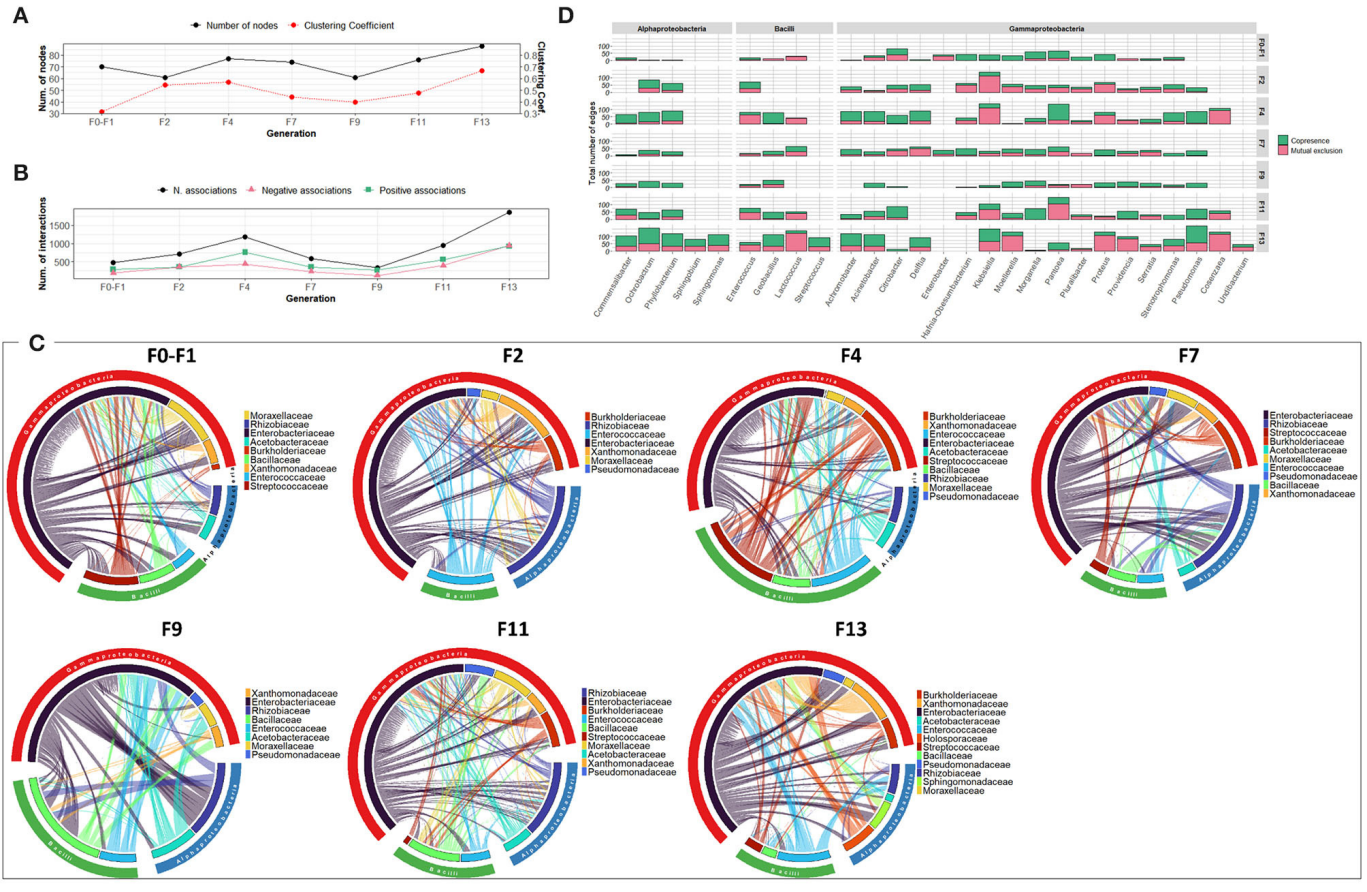
Bacterial community network within the guts of *C. capitata* adult during laboratory adaptation of Vienna 8 FD-GSS strain. **(A)** Total number of nodes and clustering coefficient, **(B)** Total number of negative (red) and positive (green) associations, **(C)** Chord diagram displaying direct relationship between OTUs at family level. The size of the ribbon is proportional to the weight of the association between the OTUs assigned to the respective segments and the color of the ribbon present in the direction of the interaction. **(D)** Number of positive and negative associations at the genus level.

Over the generations, the number of nodes contributing to the network varied slightly between 61 and 88 nodes. Fluctuation was observed in the number of associations, which varied between 383 and 1,868 associations, with an increase in the last generations (F11 and F13) (Figures 4A,B). The highest number of associations was observed between members of the *Enterobacteriaceae* family and other members of Alphaproteobacteria, mainly *Rhizobiaceae* and *Acetobacteraceae*, and members of Bacilli, mainly *Enterococcaceae* and *Bacilliaceae* (Figure 4C). Interestingly, *Pseudomonas* exhibited an increasing association over the generations despite its low relative abundance (Figure 4D, Supplementary Figure 6C).

### Comparison of Gut-Associated Bacterial Communities Over Generations at Different Developmental Stages of Laboratory-Adapted Wild Populations

The bacterial communities of larvae originating from wild populations changed dramatically during the laboratory adaptation process (PERMANOVA; *p* < 0.05) and stabilized over the F9, F11, and F13 generations (Supplementary Figures 7A,B; PERMANOVA; *p* > 0.05). In addition to the laboratory adaptation effect, the diets used over the colonization of wild larvae might enhance the differences between the generations, where separate clusters were formed based on larval diets (Supplementary Figure 7A). Proteobacteria dominated all generations (with a relative abundance of more than 90%) with the exception of F2 where a higher relative abundance of Firmicutes was observed (23.4 ± 5.2%) (Supplementary Figure 8A). At family and genus level, different bacterial composition was observed across the generations, although similar species-richness and diversity were observed across generations with a decrease in the F4 generation (Supplementary Figure 7C). The bacterial community in the F0 generation was dominated by members of *Acetobacteraceae*, mainly by *Acetobacter* (83.3 ± 6.7%) which is almost undetectable in all the other generations. Members of the *Enterobacteriaceae* family dominated F1, F2, F4, and F7 generations. *Providencia* dominated F1 and F4, *Morganella* F2, and *Klebsiella* followed by *Providencia* the F7 generation. The F9, F11, and F13 generations were mainly dominated by *Carnimonas* representing the 75.5 ± 10.6% of the bacterial community (Supplementary Figure 8C, Wilcoxon's rank sum test: *p* < 0.05).

**Figure 5:**
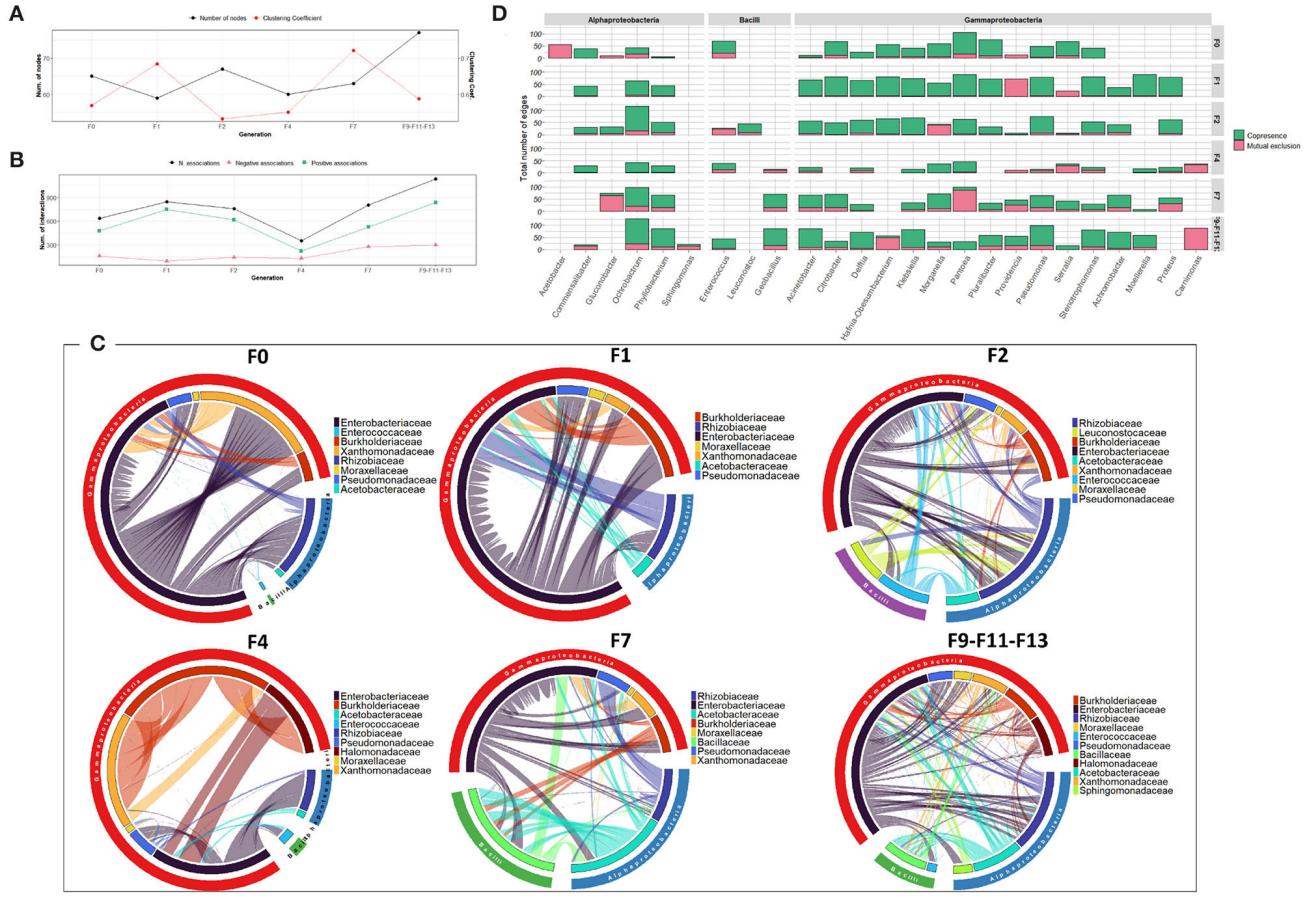
Bacterial community network within the guts of *C. capitata* larva during laboratory adaptation of wild population. **(A)** Total number of nodes and clustering coefficient. **(B)** Total number of negative (red) and positive (green) associations. **(C)** Chord diagram displaying direct relationship between OTUs at family level. The size of the ribbon is proportional to the weight of the association between the OTUs assigned to the respective segments and the color of the ribbon present in the direction of the interaction. **(D)** Number of positive and negative associations at the genus level.

Regarding the bacterial co-occurrence networks, the number of nodes forming the network varied slightly between 59 and 77 nodes (Figure 5A). The bacterial associations vary generally between 637 to 1,137 with an increase in the last generations (F9, F11, and F13). Positive associations outnumbered the negative associations in all the generations. However, an exception was observed in F4 where a reduced number of associations (353 associations) was observed which is likely due to low bacterial richness and diversity (Figure 5B). Over the generations, members of *Enterobacteriaceae* tend to be associated positively with different bacterial families to a large extent with other members of sub-phyla Gammaproteobacteria and Alphaproteobacteria, mainly the *Rhizobiaceae* family (Figures 5C,D).

As in the larval stage, the bacterial community of teneral flies changed significantly over the first generations (PERMANOVA; *p* < 0.05) and it tends to stabilize over F9, F11, and F13 (Supplementary Figures 9A,B; PERMANOVA; *p* > 0.05). Gammaproteobacteria was the prevailing class of Proteobacteria over F0, F1, F2, F4, and F7 with a relative abundance ranging from 93.3 to 99.3%. In F9, F11, and F13 generations, Gammaproteobacteria also dominated the bacterial community at a lesser degree (62.7 ± 5.6%) followed by Bacilli (16.5 ± 4.1%) and Chlamydiae (11.4 ± 3.9%) (Supplementary Figures 10A,B). Members of the *Enterobacteriaceae* family seemed to play a major role with a high abundance of different bacterial genera, such as *Klebsiella* in F0, F1, F4, F9, F11, and F13, *Pantoea* mainly in F7 and F2 generations, in addition to *Citrobacter* in F0 and F2, *Morganella* in F2 and F4, and *Providencia* mainly in F1 (Supplementary Figure 10C).

**Figure 6:**
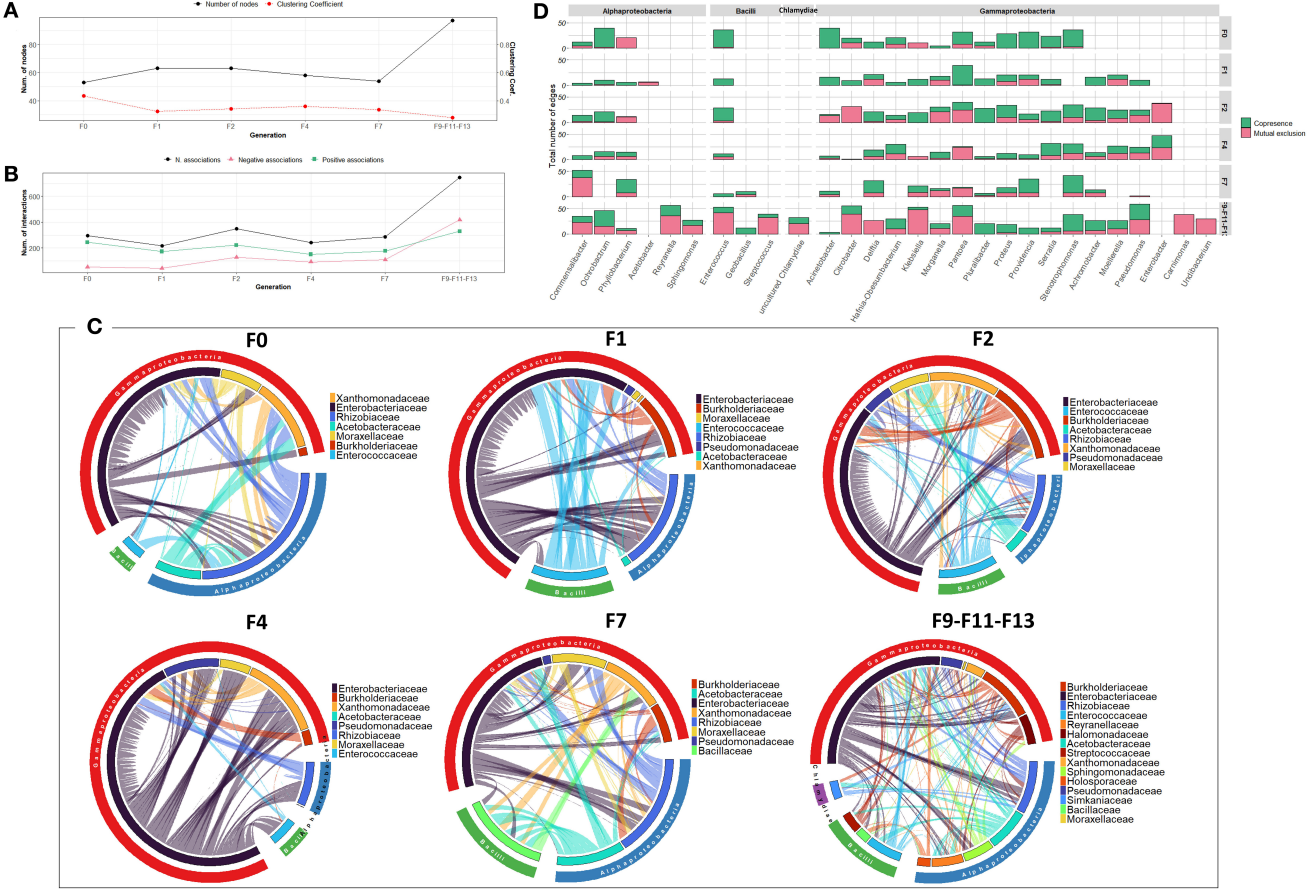
Bacterial community network within the guts of *C. capitata* teneral during laboratory adaptation of wild population. **(A)** Total number of nodes and clustering coefficient. **(B)** Total number of negative (red) and positive (green) associations. **(C)** Chord diagram displaying direct relationship between OTUs at the family level. The size of the ribbon is proportional to the weight of the association between the OTUs assigned to the respective segments and the color of the ribbon present in the direction of the interaction. **(D)** Number of positive and negative associations at the genus level.

The bacterial co-occurrence/mutual exclusion networks over the generations were characterized by a relatively narrow range in the number of nodes and associations (53–63 nodes, 216–296 associations), with an increase in the last generations F9-F11-F13 (97 nodes, 747 associations) (Supplementary Figures 6A,B). From F0 to F7 generations, members of the *Enterobacteriaceae* family were highly associated with other families of Gammaproteobacteria, as well as members of Alphaproteobacteria and Bacilli mainly with *Rhizobiaceae* and *Enterococcaceae*, respectively (Figure 6C). In the subsequent generations (F9–F11–F13), various bacterial families were contributing to the associations of the network. Interestingly, *Pseudomonas* exhibited increased associations over the generations, despite its low relative abundance (Figure 6D, Supplementary Figure 10C).

Based on CAP, NMDS, and PERMANOVA analysis, adult flies of *C. capitata* developed different bacterial profiles over the generations (Supplementary Figures 11A,B; PERMANOVA; p <0.05). The bacterial community was characterized by fluctuation in terms of species-richness and diversity across generations (Supplementary Figure 11C). Gammaproteobacteria was the most abundant class of Proteobacteria across all generations, with relative abundance ranging from 72.3 to 99.9%, followed by Alphaproteobacteria and a minor Bacilli community mainly in F7 and F13 generations (Supplementary Figures 12A,B). In Gammaproteobacteria, members of the *Enterobacteriaceae* family were dominant in all generations. *Klebsiella* dominated the bacterial communities with a relative abundance ranging from 35.7 to 98.3%. *Morganella* was significantly abundant in F2 (36.4 ± 2.8%, Wilcoxon's rank sum test: *p* < 0.05), and *Pantoea* in F7 (24.5 ± 7.2%, Wilcoxon's rank sum test: *p* < 0.05) (Supplementary Figure 12C). It is worth noting that *Enterobacter* was present mainly in adults of F2, F4, and F7 with the higher relative abundance in F2 generation (24.5 ± 7.2%, Wilcoxon's rank sum test: *p* < 0.05; Supplementary Figure 12C).

**Figure 7:**
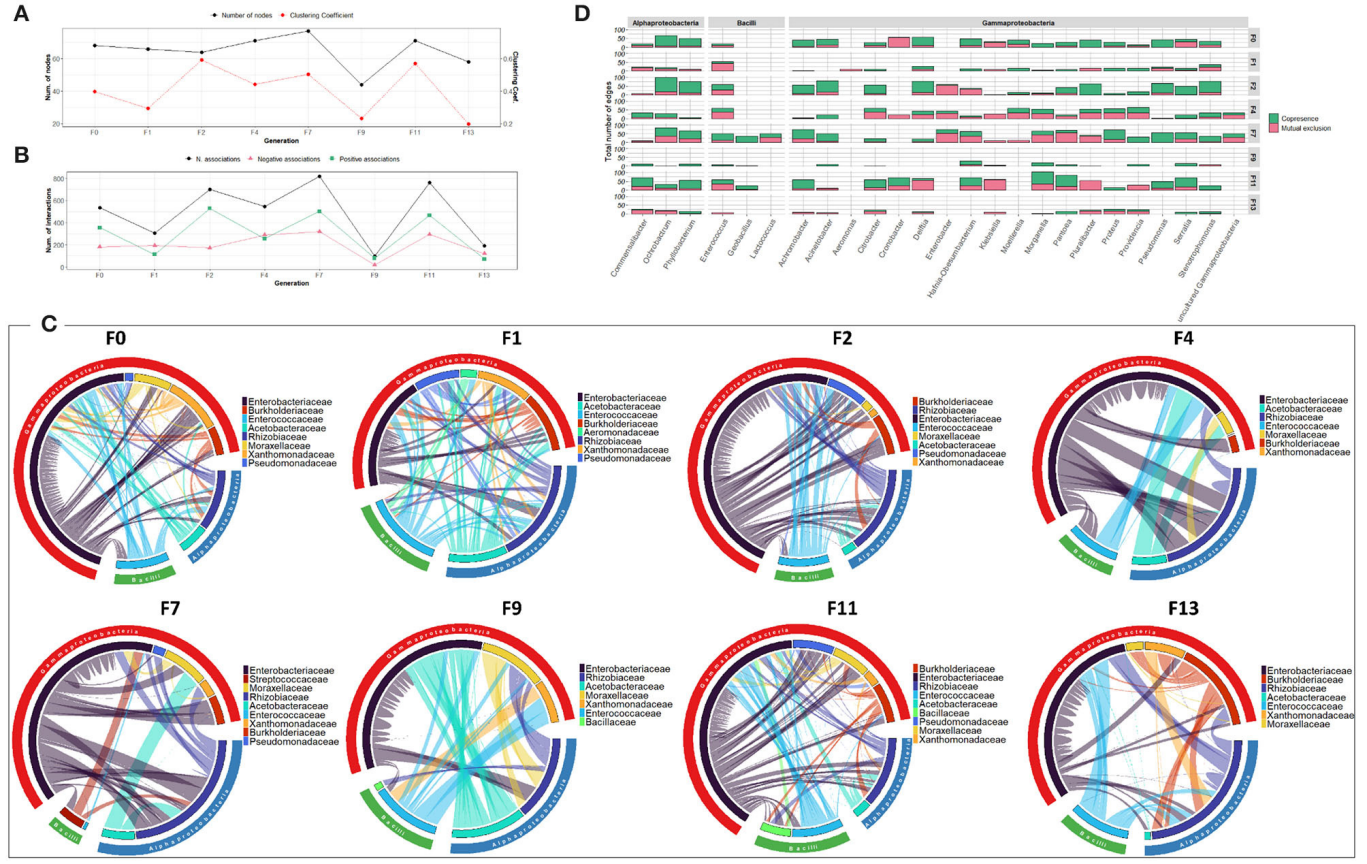
Bacterial community network within the guts of *C. capitata* adult during laboratory adaptation of wild population. **(A)** Total number of nodes and clustering coefficient. **(B)** Total number of negative (red) and positive (green) associations. **(C)** Chord diagram displaying direct relationship between OTUs at the family level. The size of the ribbon is proportional to the weight of the association between the OTUs assigned to the respective segments and the color of the ribbon present in the direction of the interaction. **(D)** Number of positive and negative associations at the genus level.

The bacterial co-occurrence/mutual exclusion networks also showed fluctuation in the number of nodes and associations ranging from 64 to 77 nodes and 307 to 819 associations with a decrease in F9 and F13 generations (44 and 58 nodes and 98 and 151 associations, respectively) (Figures 7A,B). Over the generations, members of the *Enterobacteriaceae* family were highly associated with other families of Gammaproteobacteria, as well as members of Alphaproteobacteria and Bacilli, mainly with *Rhizobiaceae* and *Enterococcaceae*, respectively (Figure 7C). At genus level, the number and type of associations across the generations varied among the different genera. The dominant genus *Klebsiella* exhibited high number of negative associations over all the generations, while *Pluralibacter* exhibited high degree of negative associations starting from F4 generation (Figure 7D).

### Convergence of Gut Bacterial Communities of Vienna 8 FD-GSS and Wild Population Over the Laboratory Adaptation Process

In order to highlight the differences and similarities among the bacterial communities associated with larvae, teneral. and adults derived from the Vienna 8 FD-GSS and the wild population, a comparison between samples from both strains at different developmental stages was performed at each generation of the laboratory adaptation process.

**Figure 8:**
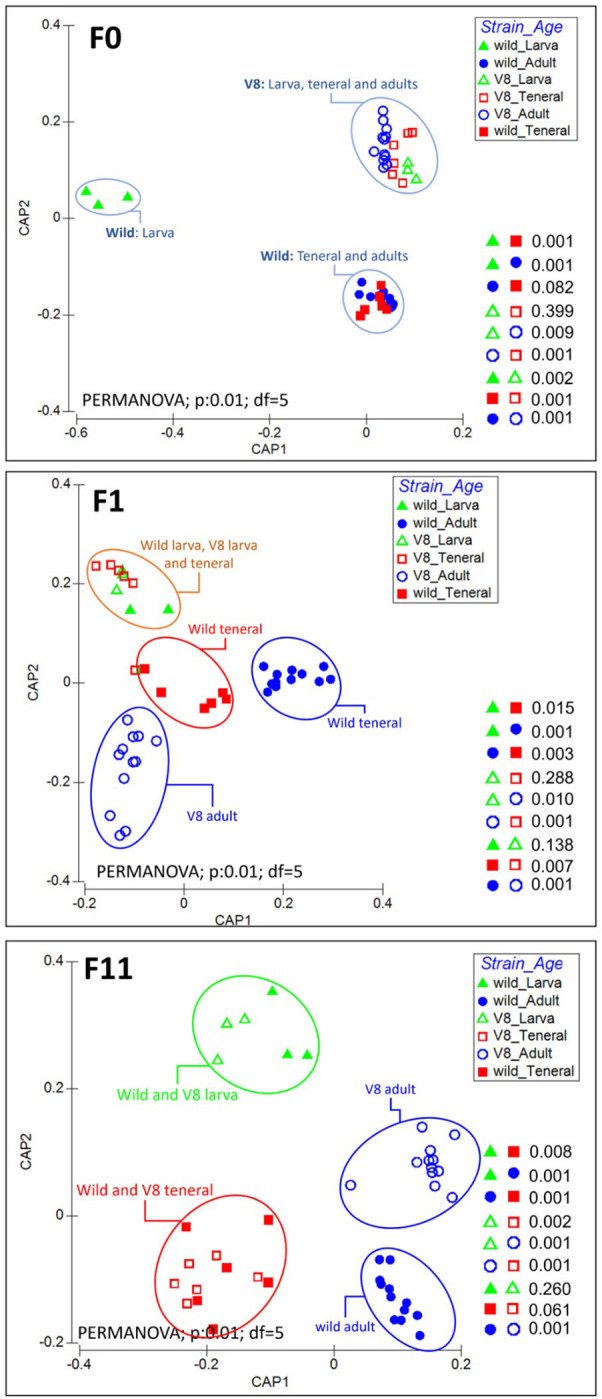
Changes in the bacterial community structure associated with the guts of larva, teneral, and adults originated from Vienna 8 FD-GSS (V8) and wild *C. capitata* stains before laboratory adaptation (F0), at F1 and F11 generations. Canonical analysis of principal coordinates (CAP) analysis and PERMANOVA test were performed based on GUniFrac dissimilarity matrix.

As evident in CAP analysis and PERMANOVA test (Figure 8, Supplementary Figure 13), during the laboratory adaptation, distinct clustering patterns were observed between flies derived from the Vienna 8 FD-GSS and the wild population at different developmental stages. Before the laboratory adaptation (F0 generation), clear clustering was observed between both strains, with all the pairwise comparisons being significantly different (Figure 8; PERMANOVA: *p* < 0.05). For Vienna 8 FD-GSS flies, similar bacterial profiles were observed between larva and teneral (PERMANOVA: p = 0.399), while, within the wild population, similarities were observed between teneral and adults (PERMANOVA: *p* = 0.082), with wild larva differentiating from all the other samples, mainly due to the predominance of *Acetobacter* (Supplementary Figure 8C). In F1 generation, the wild larva joined the Vienna 8 FD-GSS larva and teneral cluster (Figure 8; Orange circle; PERMANOVA: *p* < 0.05), while wild teneral (red circle) and adults of both strains (blue circle) continued to form a significantly different cluster (Figure 8; PERMANOVA: *p* = 0.003). During the subsequent generations, distinct clusters were observed (Supplementary Figure 13). Interestingly, after F11, the gut microbiome of flies at the same developmental stage tend to converge regardless of the origin of the strain (Figure 8), where flies originating from both Vienna 8 FD-GSS, and the wild population are clustered together at larval and teneral stages (PERMANOVA: *p* = 0.260 wild-larva/V8-larva; *p* = 0.062 wild-teneral/V8-teneral). Conversely, adults from both strains developed a significantly different bacterial profile throughout the laboratory adaptation process (Figure 8; PERMANOVA: *p* = 0.001). The bacterial diversity comparison performed based on *C. capitata* sex evidenced that there is no clear clustering between males and females (PCoA; PERMANOVA; *p* = 0.228; Supplementary Figure 14).

## Discussion

The tephritid–microbe symbiotic relationships are very intricate and of significant ecological and evolutionary importance. Expanding our knowledge of these relationships may identify ways to enhance the performance of insects that are mass- reared for SIT programs (Deutscher et al., 2019). Several studies have previously examined the gut bacterial communities associated with *C. capitata* populations. Most of these studies have shown that the bacterial composition and diversity were significantly influenced by several factors, such as laboratory adaptation, diet, rearing environment, and developmental stage (Aharon et al., 2013; Morrow et al., 2015; Malacrinò et al., 2018; Nikolouli et al., 2020). However, so far, no study has accounted for all these factors simultaneously, i.e., comparing the gut microbiome variations between wild and laboratory strain populations during colonization. In the present study, we applied a 16S rRNA amplicon sequencing approach in order to characterize the dynamics of *C. capitata* gut microbiome during laboratory adaptation of larvae and adult males and females originated from wild and Vienna 8 FD-GSS populations. As expected, our findings illustrate that during colonization, significant shifts occur in the composition and diversity of gut bacterial community of both wild and Vienna 8 FD-GSS populations, with different profiles across the developmental stages. However, no gender effect was observed.

Beta diversity analysis indicated high variability of the gut bacterial community from F0 (before laboratory adaptation) to F13. The associated bacterial communities from F7 to F13 were generally clustered separately from the previous generations. These have been observed in both strains and across the developmental stages, which indicated the “strong” effect of laboratory colonization in the alteration of the bacterial communities regardless of the strain and the age of the flies. In addition, the species-richness and diversity were generally stable across generations with a decrease in intermediate generations (at F2 for all the life stages of Vienna 8 FD-GSS and F4, F7 and F9 for wild larva, teneral, and adults, respectively); this finding indicated a turnover and/or reorganization of the bacterial community under artificial rearing conditions at specific generations. Beside the laboratory adaptation, we evidenced that the gut microbiota community structure of *C. capitata* shifts during developmental stages: larvae, teneral, and adults (including 5 and 15 days). This finding was in line with previous studies performed on *C. capitata* populations (Aharon et al., 2013; Malacrinò et al., 2018). To the best of our knowledge, the effect of laboratory adaptation was investigated only in two tephritid flies: *Anastrepha fraterculus* (Salgueiro et al., 2020) and *Bactrocera tryoni* (Majumder et al., 2022). Both studies using 16S rRNA gene amplicon data sets reported a strong effect of the degree of laboratory adaptation on the structure of the gut bacterial community at each developmental stage. In addition, Salgueiro et al. (2020) also reported that after six generations under artificial rearing conditions, the wild laboratory-adapted *A. fraterculus* harbor a different gut bacterial community compared to the long established colony (Salgueiro et al., 2020). The gut bacterial community is dynamic and could be altered by diet and rearing environment conditions as previously reported in the genera of different flies including *Drosophila***. ***melanogaster* (Staubach et al., 2013), *Drosophila suzikii* (Jiménez-Padilla et al., 2020), and *Zeugodacus cucurbitae* (Asimakis et al., 2019).

The bacterial community associated with the studied populations of *C*. *capitata* was mainly composed by members of Proteobacteria (>87% of reads) particularly by Gammaproteobacteria; followed by Firmicutes, and to a lesser extent, by the members of Chlamydiae. In Gammaproteobacteria, members of the family, *Enterobacteriaceae* dominated the bacterial community of wild and laboratory populations of *C. capitata* flies representing more than 69% of the total bacterial community. The occurrence of members of *Enterobacteriaceae* in high abundance was consistent with what has been reported previously on *C. capitata* (Behar et al., 2008a,b,c; Aharon et al., 2013; De Cock et al., 2019) and most of the studies on tephritid flies (Morrow et al., 2015; Yong et al., 2017; Augustinos et al., 2019; De Cock et al., 2020; Raza et al., 2020; Salgueiro et al., 2020). Morphological characteristics and behavior of fruit flies, which contribute to both vertical and horizontal transmission of *Enterobacteriaceae*, suggests that these bacteria play an important role in fruit fly development and physiology (Deutscher et al., 2019). Comparing the bacterial community at genus level, we found that the most abundant bacteria in the laboratory-adapted Vienna 8 FD-GSS population were *Providencia* mainly in F0, F1, and F2 generations. This genus has been commonly detected in Vienna 8 FD-GSS and other laboratory colonies (Ben Ami et al., 2010; Augustinos et al., 2015; De Cock et al., 2019). However, in subsequent generations we observed a reorganization of the bacterial community depending on different developmental stages, where the larval stage was characterized by high abundance of *Carnimonas* (from F4 to F13), *Pluralibacter*, and *Klebsiella* in teneral (F4 and F7) and adults (F7 and F9). Interestingly, members of Bacilli class (Firmicutes phylum), mainly *Lactococcus* (F4, F7, and F13) and *Enterococcus* (F9 and F11) were highly present in Vienna 8 FD-GSS adults. Firmicutes have not been frequently reported in *C. capitata* populations, although Malacrinò et al. (2018) using 16S rRNA gene amplicon, have recently related the presence of members of Firmicutes, such as *Lactococcus* and *Leuconostoc*, with the developmental stage and diet. However, they were not highly present in adults (<2%). Conversely, members of Bacilli class were more common in most laboratory reared adults of *Bactrocera* sp. such as *B*. *tryoni* (Morrow et al., 2015), *B. dorsalis* (Andongma et al., 2015; Stathopoulou et al., 2019), and *B. oleae* (Estes et al., 2012). Moreover, we cannot exclude the possibility that some of the less frequent bacteria are actually members of the insect exterior and not members of the gut bacteriome.

The populations of wild teneral and adults seem to be more resistant to artificial rearing conditions compared to the Vienna 8 FD-GSS colony. The effect of laboratory adaptation was slightly observed within the wild population, where similar patterns were observed in the gut microbiome across the thirteen generations, with the dominance of the *Enterobacteriaceae* family. At genus level, across all generations, the teneral and adult flies were mainly dominated by *Klebsiella*, followed by *Pantoea* in teneral and *Pluralibacter* in adults. *Klebsiella* has been reported as the dominant symbiont of most wild *C. capitata* populations (Behar et al., 2005; Augustinos et al., 2015; Nikolouli et al., 2020). In contrast to the stages of teneral and adults, the gut microbiota of wild larvae was strongly associated with the diets and degree of laboratory adaptation. Initially, the larvae collected from mandarin fields (F0) were dominated by *Acinetobacter*; during the first generation of laboratory adaptation (F1, F2, F4, and F7), members of the *Enterobacteriaceae* family dominated the larvae raised on mango, while *Carnimonas* dominated F9, F11, and F13 generations fed on artificial diet. The diet-related effect was previously explained in *C. capitata* larvae (Malacrinò et al., 2018). However, a direct comparison can be difficult to perform due to multiple factors, citing mainly the different diets used (figs, prickly pears, peaches, cherimoya, and orange fruits, compared to mandarin and mango used in our study), the strain and the origin of the population (either laboratory colony or wild populations).

A better understanding of the core (ubiquitous and abundant) bacteria during colonization of *C. capitata* population is critical to manipulate the associated bacterial communities under artificial rearing condition for the functional improvement of SIT application. Our overall core taxa analysis evidenced nine taxa (*Klebsiella, Providencia, Citrobacter, Pluralibacter, Pantoea* (2 variants), *Pseudomonas, Phyllobacterium*, and *Enterococcus*) that were conserved under artificial rearing condition. As previously shown in this study and other studies, the gut of wild and Vienna 8 FD-GSS population differs in terms of bacterial composition and abundance which affects the dynamics of the *C. capitata* bacteriome during the laboratory adaptation. Thus, in our investigation, we separated the core microbiome for each strain and developmental stage. This approach provides three key insights on the dynamics of the core microbiome through the laboratory adaptation process; first, the core members were conserved through the laboratory adaptation; second, certain taxa that joined the core microbiome at a particular generation were maintained through the rest of the generations, and third, taxa that were part of the core microbiome during the first generations were lost during the laboratory adaptation. The detailed characterization of the core microbiome in medfly will enable research groups (i) to better understand the effect of the radiation on the medfly bacteriome and (ii) provide strategies for the restoration of the core microbiome after radiation e.g., the use of specific bacterial strains as probiotics and specific diets.

Core microbiome taxa with a reported beneficial effect include some strains of *Klebsiella, Citrobacter*, and *Pantoea* that are mostly related to nitrogen fixation. These diazotrophic bacteria provide their hosts with the nitrogen resources required, that are otherwise unable to receive from their nitrogen-poor diet (Ohkuma et al., 1999; Lauzon, 2003; Behar et al., 2005). Using *Klebsiella* sp. as a supplement in larval and adult diets has been proven to affect different parameters of rearing and biological quality of released males (Ben Ami et al., 2010; Hamden et al., 2013; Augustinos et al., 2015). Our analysis indicated that *Klebsiella* was the common genus shared between 99.6% of the total samples of both strains and during all the generations with higher abundance in wild population compared to Vienna 8 FD-GSS populations. In addition to the members of the *Enterobactericeae* family, *Enterococcus* sp. (*Enterococcaceae*) was also reported to be a beneficial species for *B. dorsalis* GSS populations, where the flies fed on diets enriched with this bacterium had a reduced larval developmental duration and higher pupal weight and percentage of survival (Khaeso et al., 2018; Zhang et al., 2021). Correspondently, *Enterococcus* were broadly present mainly in teneral and adults of Vienna 8 FD-GSS population across all the generations, while they were acquired at F7 in larval stage. In contrast, *Enterococcus* were under the detection level in wild population due to their low relative abundance. Interestingly, pathogenic bacteria, such as *Pseudomonas* were also detected as a part of the core microbiome in almost all the generations for both strains. Despite the high prevalence of *Pseudomonas*, it was highly abundant mainly in the last generations of the Vienna 8 FD-GSS colony evolution (F4 for larvae, F9 and F11 for teneral, and F13 for adults). Similar phenomenon (but with lower abundance) was observed in the wild population. Accordingly, these results evidenced that the laboratory adaptation enhanced to some extent the development of a *Pseudomonas* community within *C. capitata* populations, which is probably due to the decrease of the *Enterobacteriaceae* family, mainly *Klebsiella*, in the last generation. These findings were supported by previous studies, which indicated that the enrichment of *C. capitata* diet with *Klebsiella* sp. decreased the levels of *Pseudomonas* sp. (Behar et al., 2008c; Ben Ami et al., 2010). On the other hand, among the taxa that joined the core microbiome during laboratory adaptation, *Carnimonas* were acquired by both wild and Vienna 8 FD-GSS larvae (at F9 and F4, respectively) with high relative abundance but were completely absent in teneral and adult stages. This is the first report of *Carnimonas* in *C. capitata* populations and in tephritid, in general. In contrast, it had previously been reported as persistent in a mass rearing colony and a wild population of *Anagyrus vladimiri* but with low abundance (<1%) (Izraeli et al., 2021). However, higher abundance of *Carnimonas* (32%) was recently identified in *Hyalomma anatolicum* ticks; meanwhile, the authors suggested a negative association between *Klebsiella* and *Carnimonas* (Perveen et al., 2022). So far, little is known about the role of *Carnimonas*. The understanding of the core microbiome composition and variation during different stages of laboratory adaptation, would allow to design a population-specific rearing protocol that would either maintain key players in the microbiome of the colonized flies or restore them as a part of the rearing process. For instance, *Enterobacter* is considered as a key member of *C. capitata* gut bacterial community mainly in laboratory colonies (Augustinos et al., 2015; Kyritsis et al., 2017). However, in the present study, low relative abundance of *Enterobacter* was observed across the developmental stages/generations, which may negatively affect the fitness and the biological quality of the reared flies. Thus, the use of *Enterobacter* as a probiotic supplement could be exploited to enhance the production of high-quality flies and therefore, increase the effectiveness of the SIT (Hamden et al., 2013; Augustinos et al., 2015; Kyritsis et al., 2019).

We further investigated the variation of the bacterial associations within the gut of *C. capitata* populations through the laboratory colonization. Overall, we estimated a high number of interactions in the last generation including F9, F11, and F13 of both strains of *C. capitata* flies compared to the previous generations which reflect, to some extent, the equal contribution of different bacterial taxa in the formation of the community and therefore the stability of the bacterial community (i.e., robustness of the community against external alterations). Interestingly, certain taxa with lower relative abundance, such as *Serratia, Stenotrophomonas, Delftia, Proteus Ochrobacterum*, and *Commemsalibacter* maintain high number of interactions over the generations for the three studied developmental stages, suggesting that even low abundant taxa may play an important role in shaping the total bacterial community. Over generations, most of the association were mostly driven by members of *Enterobacteriaceae*, where they interact mainly with other members of Gammaproteobacteria, while outside Gammaproteobacteria, they interact mainly with the members of *Rhizobiaceae*.

In this study, we identified for the first time, sequences belonging to an uncultured Chlamydiae (*Simkaniaceae*). The presence of these bacteria was restricted to the last generations of the laboratory adaptation process (from F4 onwards) with a relative abundance ranging from 0.2 to 23.9%. The higher abundance was observed in wild teneral files mainly at F9 and F11 generations, representing respectively 23.9 and 10.2% of the total bacterial community. Since Chlamydiae phylum has never been identified in either wild or laboratory *C. capitata* populations, we suggest that it was acquired from the surrounding environment of the laboratory-adapted flies. A limited number of studies reported the presence of Chlamydiia communities in insects and other arthropods, with either low abundance, such as in *Heliconius* butterflies (Schooten et al., 2018) or high, such as in *Dermacentor marginatus* ticks (Zhang et al., 2019). To the best of our knowledge, within insects, it was reported in *Heliconius* butterflies (Schooten et al., 2018), the whitefly *Bemisia tabaci* (Thao et al., 2003) and with pathogenic role in the cockroach *Bombina orientalis* (Corsaro et al., 2007). Furthermore, members of Chlamydiae including *S. negevensis* (*Simkaniaceae* family), *Chlamydia trachomatis*, and *C. pneumoniae* (*Chlamydiaceae* family) have been previously reported as pathogenic for humans and animals (Karlsen et al., 2008; Nascimento-Carvalho et al., 2009; Baud et al., 2015). This finding may shed a new light on the possible symbionts and/or pathogens that could be transmitted from the mass rearing facilities and thus, impact the efficiency of the SIT application.

## Conclusion

This investigation settled an additional cornerstone in the characterization of the bacterial community of gut microbiome and the core microbiome associated with a laboratory-adapted *C. capitata* population dedicated for SIT application. In the present study, we evidenced that the laboratory adaptation strongly affects the bacterial community structure of both wild and Vienna 8 FD-GSS populations. Moreover, *C. capitata* flies harbor different intestinal microbiome across the developmental stages. Interestingly, over the generations, a convergence between the gut bacterial community associated with the wild and the Vienna 8 FD-GSS populations at each developmental stage was revealed, which might unravel the ability of colonization (i.e., rearing condition and diet) to “homogenize” the gut microbiome of both stains. Understanding the dynamics of the bacterial community during laboratory adaptation, may provide guidance for its efficient manipulation, in such a manner, which would allow for the maintenance or introduction of selected symbionts in order to improve the physiology and behavior of the reared flies, and therefore produce better performing and cost-effective flies for a successful SIT application.

## Data Availability Statement

The datasets presented in this study can be found in online repositories. The names of the repository/repositories and accession number(s) can be found below: https://www.ncbi.nlm.nih.gov/, PRJNA822871.

## Author Contributions

GT and OD designed the study. NB, MC-O, PS, EA, and IR performed the experiments. NB, MC-O, PS, EA, OD, JG, AM, MB, NR, and GT interpreted and analyzed data. NB prepared the figures. NB and MC-O wrote the first draft of the manuscript. EA, PS, OD, and GT critically revised the manuscript. All authors reviewed and approved the final version of the manuscript.

## Funding

The present study was supported by the Joint FAO/IAEA Insect Pest Control Subprogramme through the CRP project Improvement of Colony Management in Insect Mass-rearing for SIT Applications and a research contract entitled, Uncovering the Unknown Insect Virome, Bacterial Micro-Diversity and Fungal-Biome (No22662). This research has been partially supported with the support of the Erasmus+ Programme of the European Union.

## Conflict of Interest

MC-O and JG were employed by Empresa de Transformación Agraria S.A., S.M.E., M.P. (TRAGSA).

The remaining authors declare that the research was conducted in the absence of any commercial or financial relationships that could be construed as a potential conflict of interest.

## Publisher's Note

All claims expressed in this article are solely those of the authors and do not necessarily represent those of their affiliated organizations, or those of the publisher, the editors and the reviewers. Any product that may be evaluated in this article, or claim that may be made by its manufacturer, is not guaranteed or endorsed by the publisher.
