# Supplementary material for: Dynamics of the Gut Bacteriome During a Laboratory Adaptation Process of the Mediterranean Fruit Fly, Ceratitis capitata
Source: Front Microbiol. 2022 Jul 1;13:919760. doi: 10.3389/fmicb.2022.919760 (PMC9283074; doi:10.3389/fmicb.2022.919760)
Supplement: Supplementary file 1 [file Data_Sheet_1.zip › Supplementary Material/Supplementary Figures.docx]

Supplementary Material

**Supplementary Table 1.** Dietary of larval and adults for each generation and strain

| **Generation** | **Vienna8-GSS** | | | | | | | **Wild** | | | | | | | **Total samples** |
| --- | --- | --- | --- | --- | --- | --- | --- | --- | --- | --- | --- | --- | --- | --- | --- |
|  | **Larva*** | **Teneral**** | | **Adult**** | | | | **Larva*** | **Teneral**** | | **Adult**** | | | |  |
|  |  |  |  | **5 days** | | **15 days** | |  |  |  | **5 days** | | **15 days** | |  |
|  |  | **M** | **F** | **M** | **F** | **M** | **F** |  | **M** | **F** | **M** | **F** | **M** | **F** |  |
| **F0** | 3x5 ^a^ | 3x5 | 3x5 | 3x5 | 3x5 | 3x5 | 3x5 | 3x5 ^d^ | 3x5 | 3x5 | 3x5 | 3x5 | 3x5 | 3x5 | 42 |
| **F1** | 3x5 ^b^ | 3x5 | 3x5 | 3x5 | 3x5 | 3x5 | 3x5 | 3x5 ^c^ | 3x5 | 3x5 | 3x5 | 3x5 | 3x5 | 3x5 | 42 |
| **F2** | 3x5 ^b^ | 3x5 | 3x5 | 3x5 | 3x5 | 3x5 | 3x5 | 3x5 ^c^ | 3x5 | 3x5 | 3x5 | 3x5 | NC | NC | 36 |
| **F4** | 3x5 ^b^ | 3x5 | 3x5 | 3x5 | 3x5 | 3x5 | 3x5 | 3x5 ^c^ | 3x5 | 3x5 | 3x5 | 3x5 | 3x5 | 3x5 | 42 |
| **F7** | 3x5 ^b^ | 3x5 | 3x5 | 3x5 | 3x5 | 3x5 | 3x5 | 3x5 ^c^ | 3x5 | 3x5 | 3x5 | 3x5 | 3x5 | 3x5 | 42 |
| **F9** | 3x5 ^c^ | 3x5 | 3x5 | 3x5 | 3x5 | 3x5 | 3x5 | 3x5 ^c^ | 3x5 | 3x5 | 3x5 | 3x5 | 3x5 | 3x5 | 42 |
| **F11** | 3x5 ^c^ | 3x5 | 3x5 | 3x5 | 3x5 | 3x5 | 3x5 | 3x5 ^c^ | 3x5 | 3x5 | 3x5 | 3x5 | 3x5 | 3x5 | 42 |
| **F13** | 3x5 ^c^ | NC | NC | 3x5 | 3x5 | 3x5 | 3x5 | 3x5 ^c^ | 3x5 | 3x5 | 3x5 | 3x5 | 3x5 | 3x5 | 36 |
| **Total samples** | 24 | 21 | 21 | 24 | 24 | 24 | 24 | 24 | 24 | 24 | 24 | 24 | 21 | 21 | **324** |

3x5: three replicates x five individuals per replicate

NC: Samples not collected

*:3^rd^ instar larva

a: Larva fed on mandarin fruit

b: Larva fed on mango fruit

c: Larva fed on artificial diet (Caudete)

d: Larva fed on artificial diet (Seibersdorf)

**: Teneral and adult flies fed on a mixture of sugar, yeast, and water

**
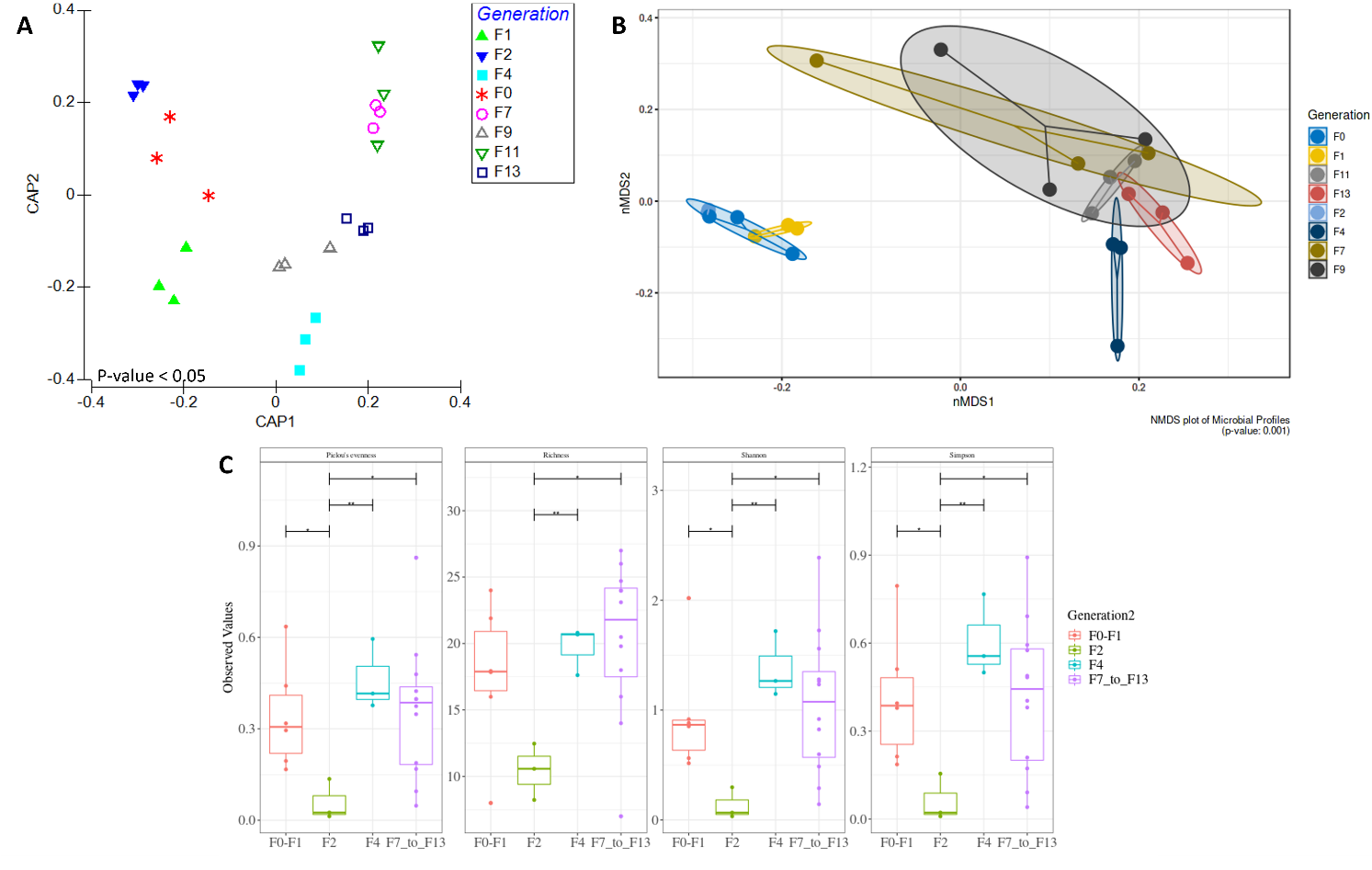
**

**Supplementary Figure 1.** Diversity of bacterial communities derived from guts of *C. Capitata* larva during laboratory adaptation of Vienna8FD-GSS strain. CAP **(A)** and NMDS **(B)** analyses were used to find axes that best discriminate the groups of interest. **(C)** Species richness and diversity indices with significance differences, boxes represent the interquartile range (IQR), the line within the boxes is the median, and the dots represent samples.


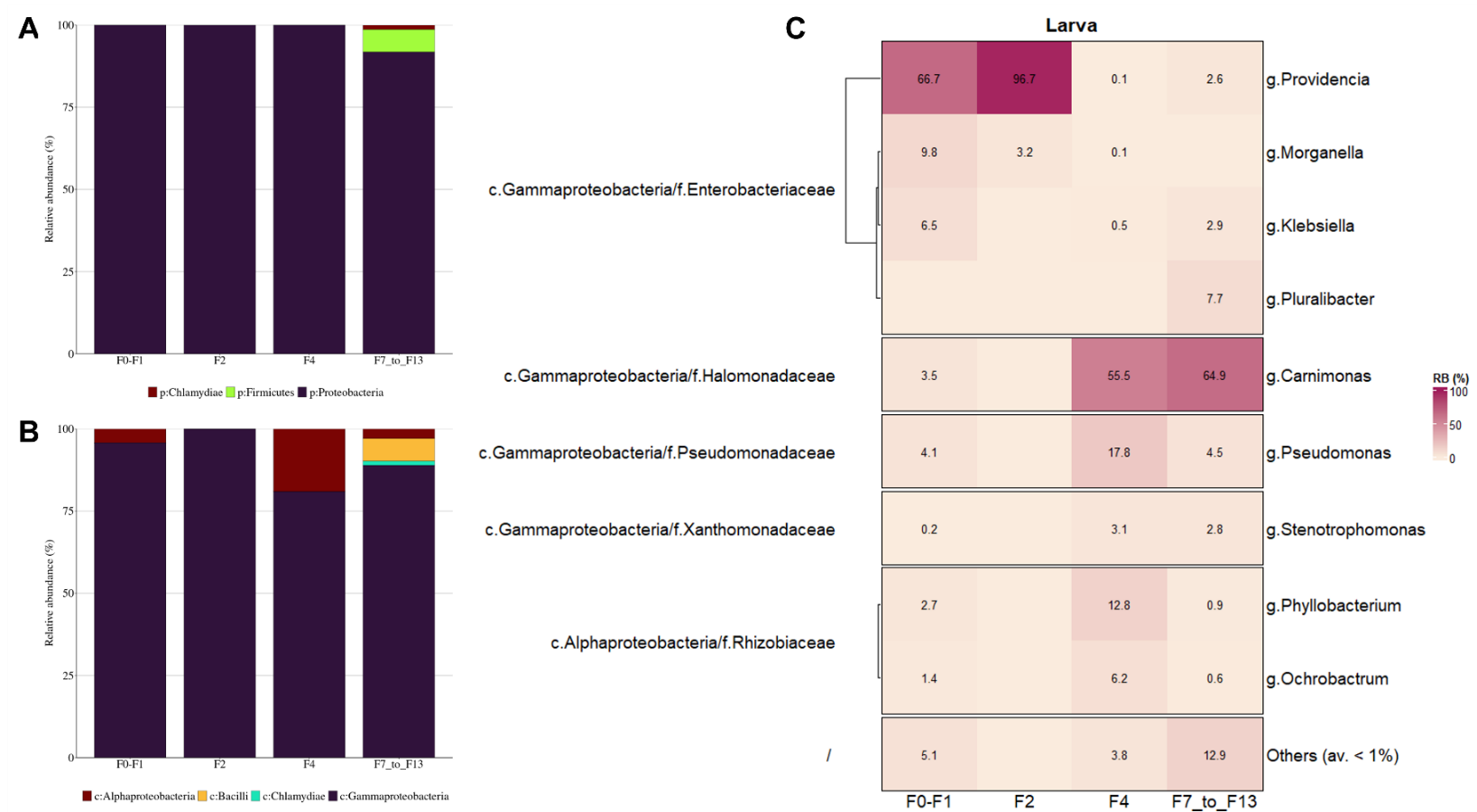


**Supplementary Figure 2.** Composition of bacterial community associated to guts of *C. Capitata* larva during laboratory adaptation of Vienna 8-GSS strain. **(A)** Phylum level, **(B)** Class level and **(C)** Genus level.


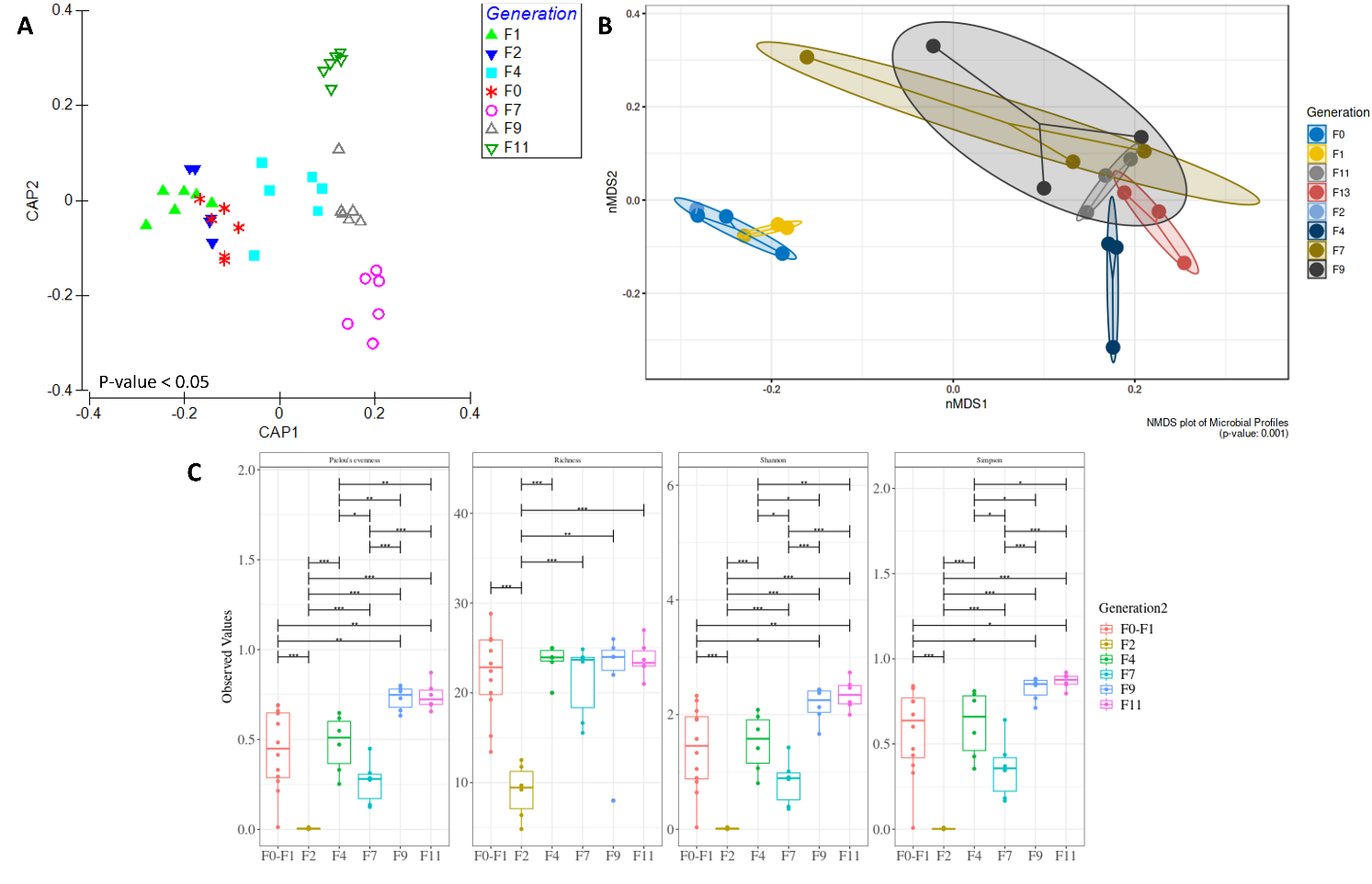


**Supplementary Figure 3**: Diversity of bacterial communities derived from guts of C. Capitata teneral during laboratory adaptation of Vienna8FD-GSS strain. CAP **(A)** and NMDS **(B)** analyses were used to find axes that best discriminate the groups of interest. **(C)** Species richness and diversity indices with significance differences, boxes represent the interquartile range (IQR), the line within the boxes is the median, and the dots represent samples.


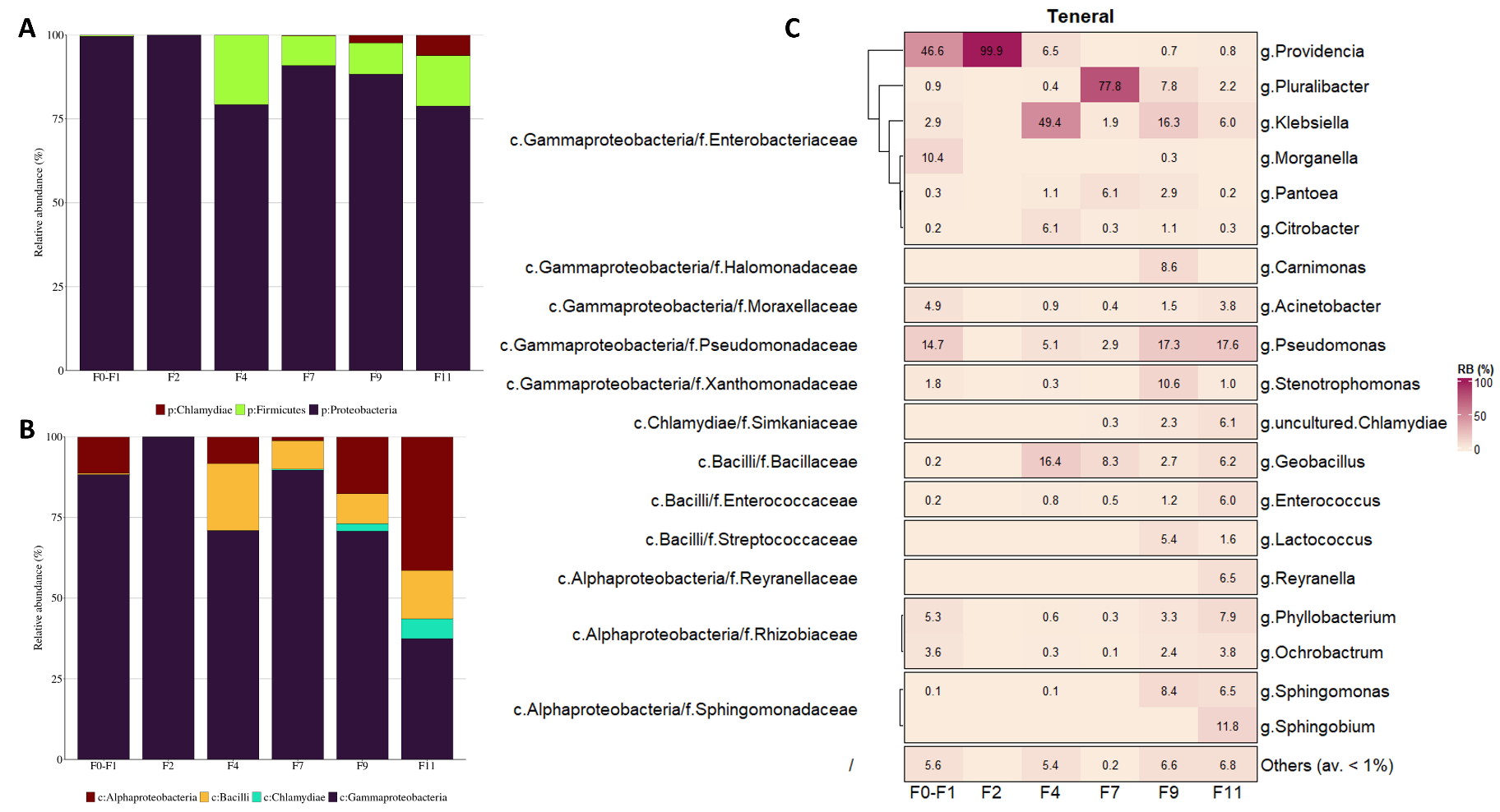


**Supplementary Figure 4:** Composition of bacterial community associated to guts of *C. Capitata* teneral during laboratory adaptation of Vienna8FD-GSS strain. **(A)** Phylum level, **(B)** Class level and **(C)** Genus level.


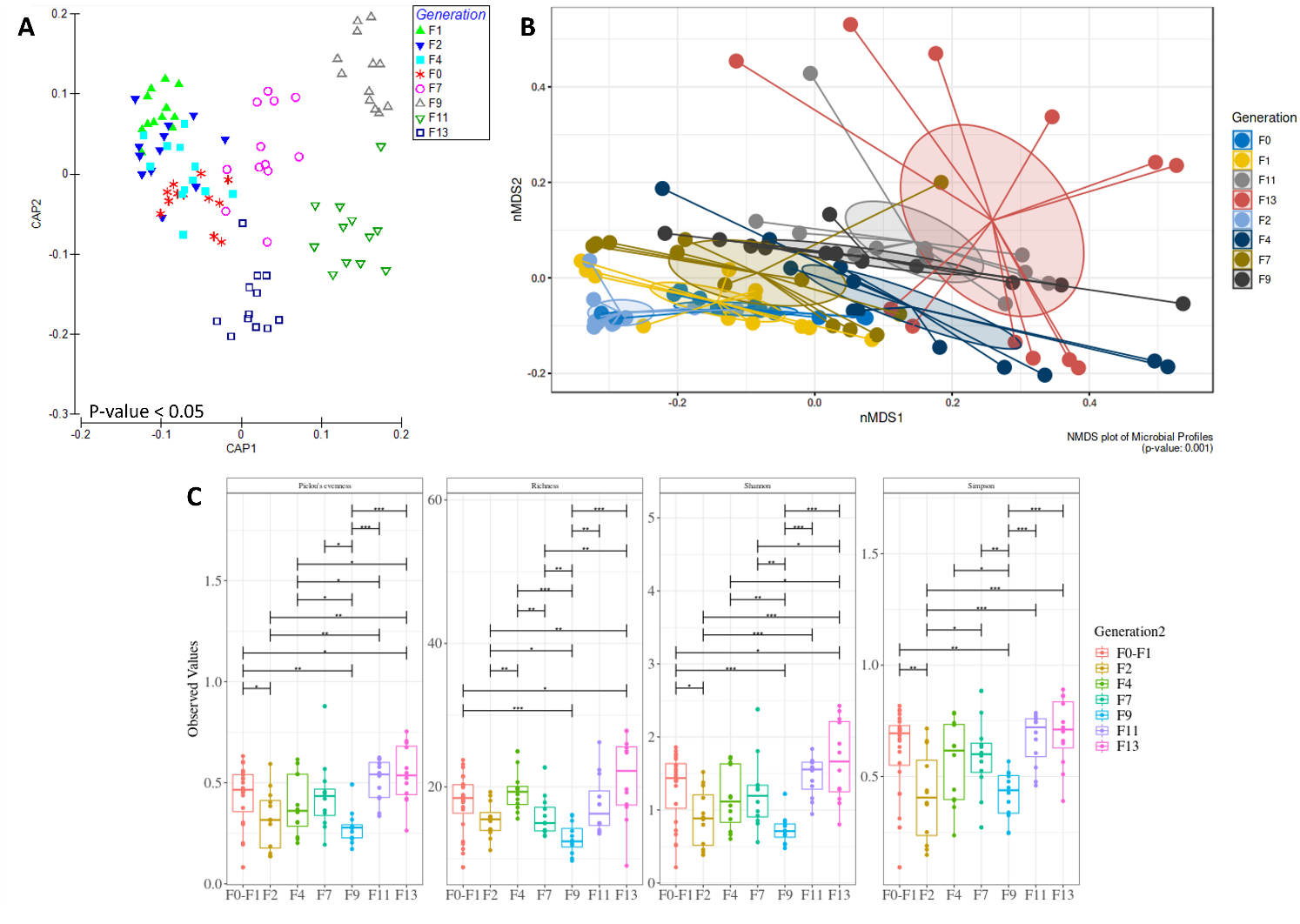


**Supplementary Figure 5:** Diversity of bacterial communities derived from guts of *C. Capitata* adult during laboratory adaptation of Vienna8FD-GSS strain. CAP **(A)** and NMDS **(B)** analyses were used to find axes that best discriminate the groups of interest. **(C)** Species richness and diversity indices with significance differences, boxes represent the interquartile range (IQR), the line within the boxes is the median, and the dots represent samples.


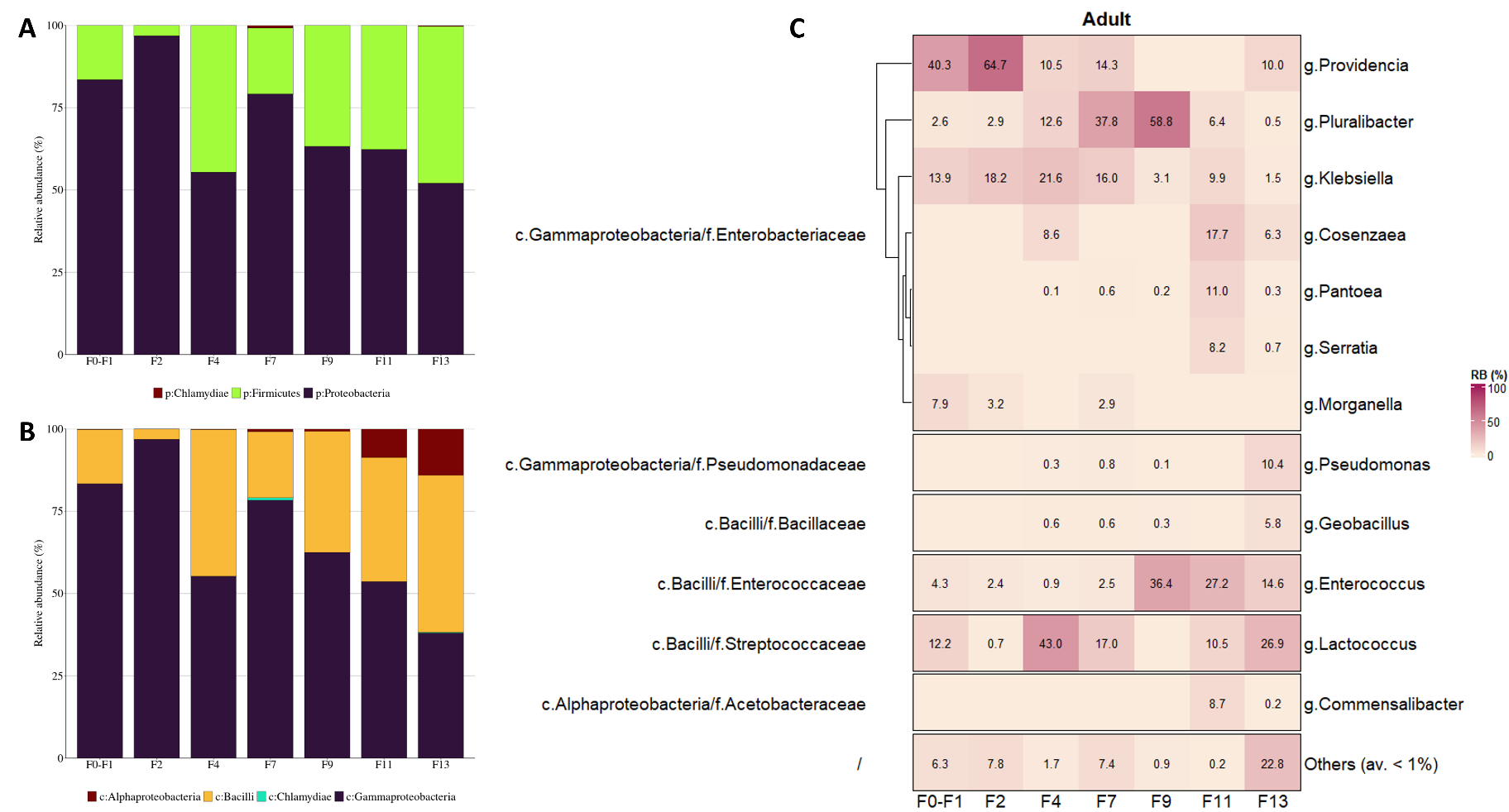


**Supplementary Figure 6:** Composition of bacterial community associated to guts of *C. Capitata* adult during laboratory adaptation of Vienna8FD-GSS strain. **(A)** Phylum level, **(B)** Class level and **(C)** Genus level.


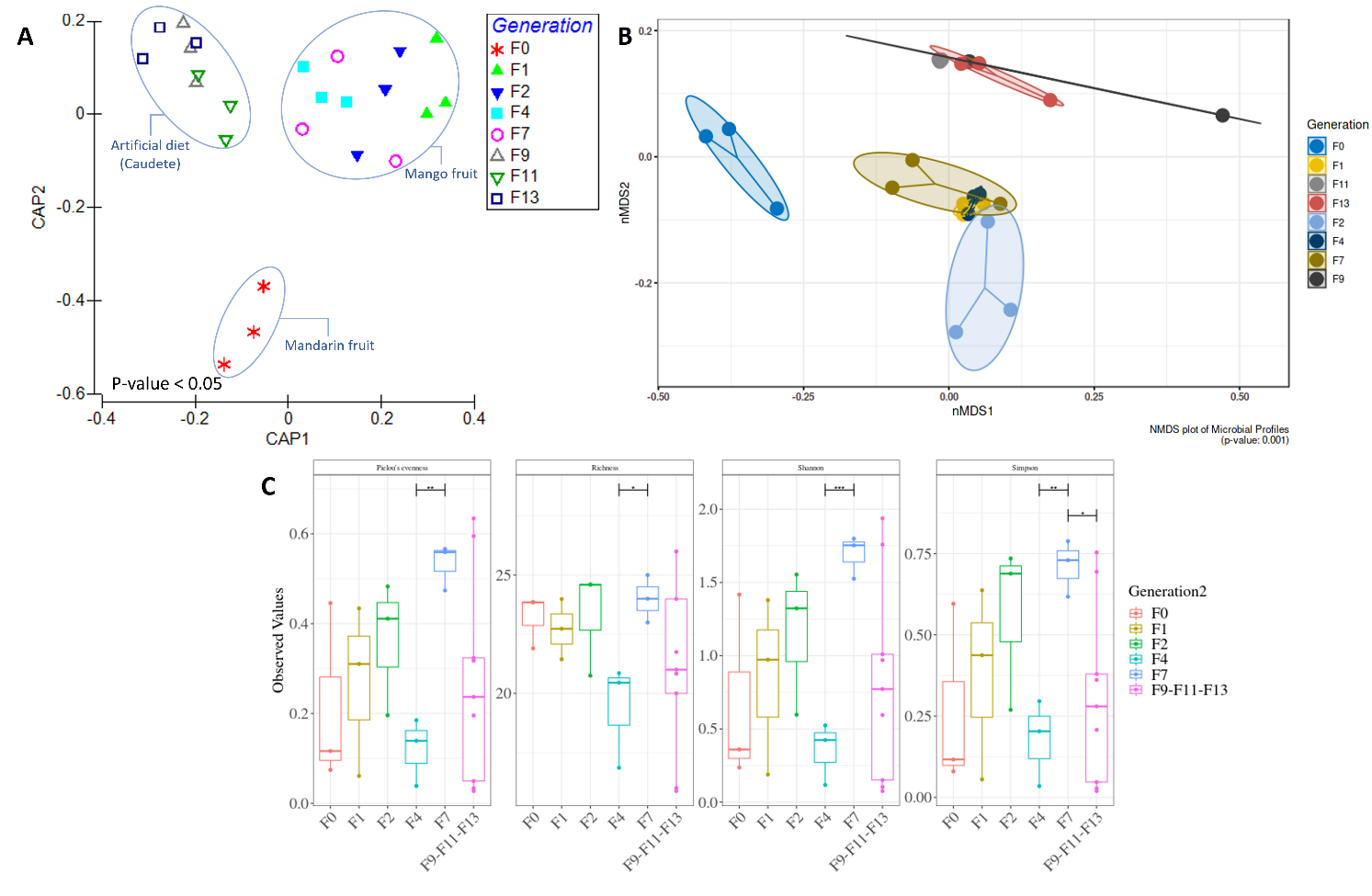


**Supplementary Figure 7:** Diversity of bacterial communities derived from guts of *C. Capitata* larva during laboratory adaptation of the wild population. CAP **(A)** and NMDS **(B)** analyses were used to find axes that best discriminate the groups of interest. **(C)** Species richness and diversity indices with significance differences, boxes represent the interquartile range (IQR), the line within the boxes is the median, and the dots represent samples.


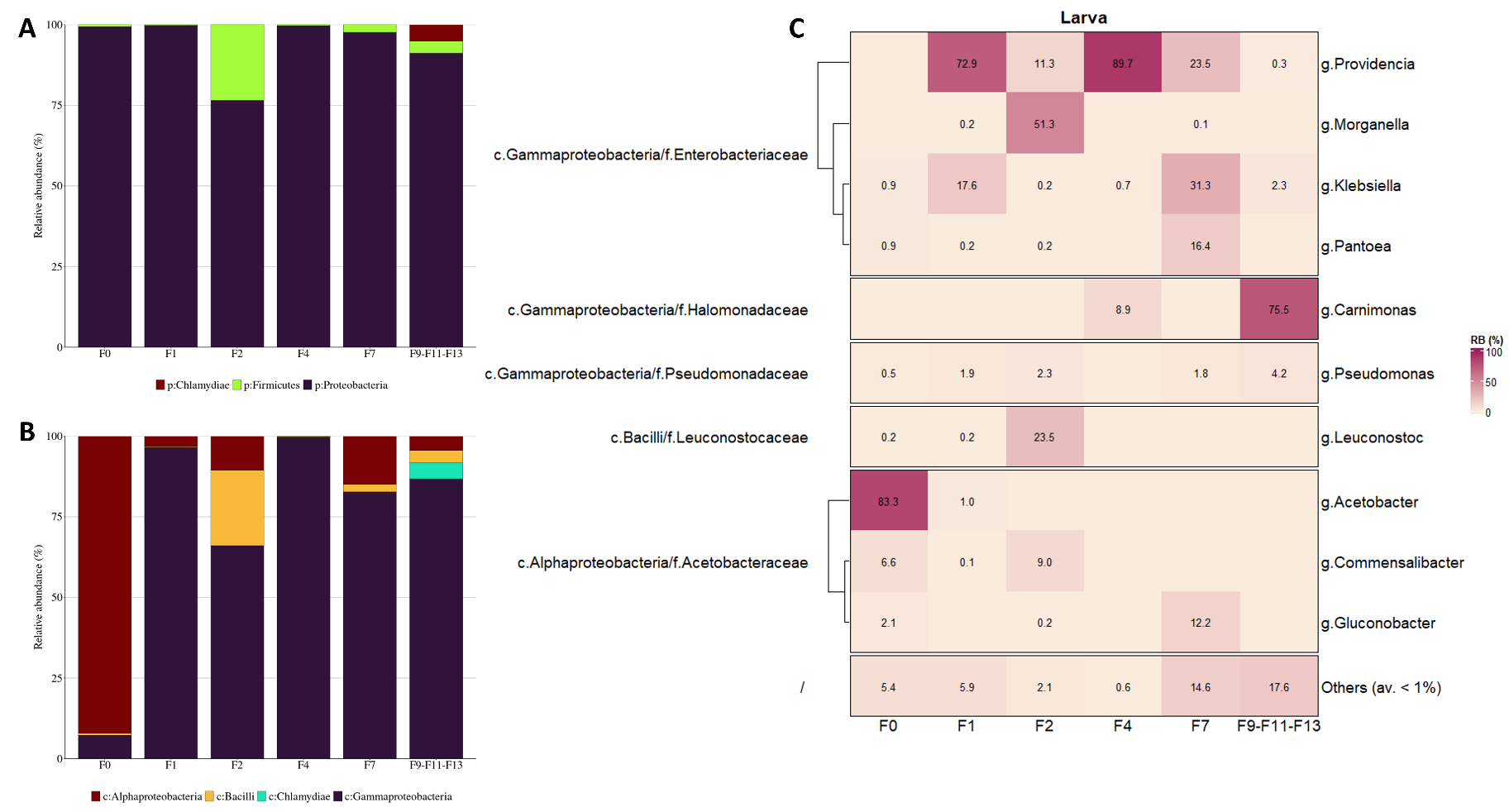


**Supplementary Figure 8:** Composition of bacterial community associated to guts of *C. Capitata* larva during laboratory adaptation of wild population. **(A)** Phylum level, **(B)** Class level and **(C)** Genus level.


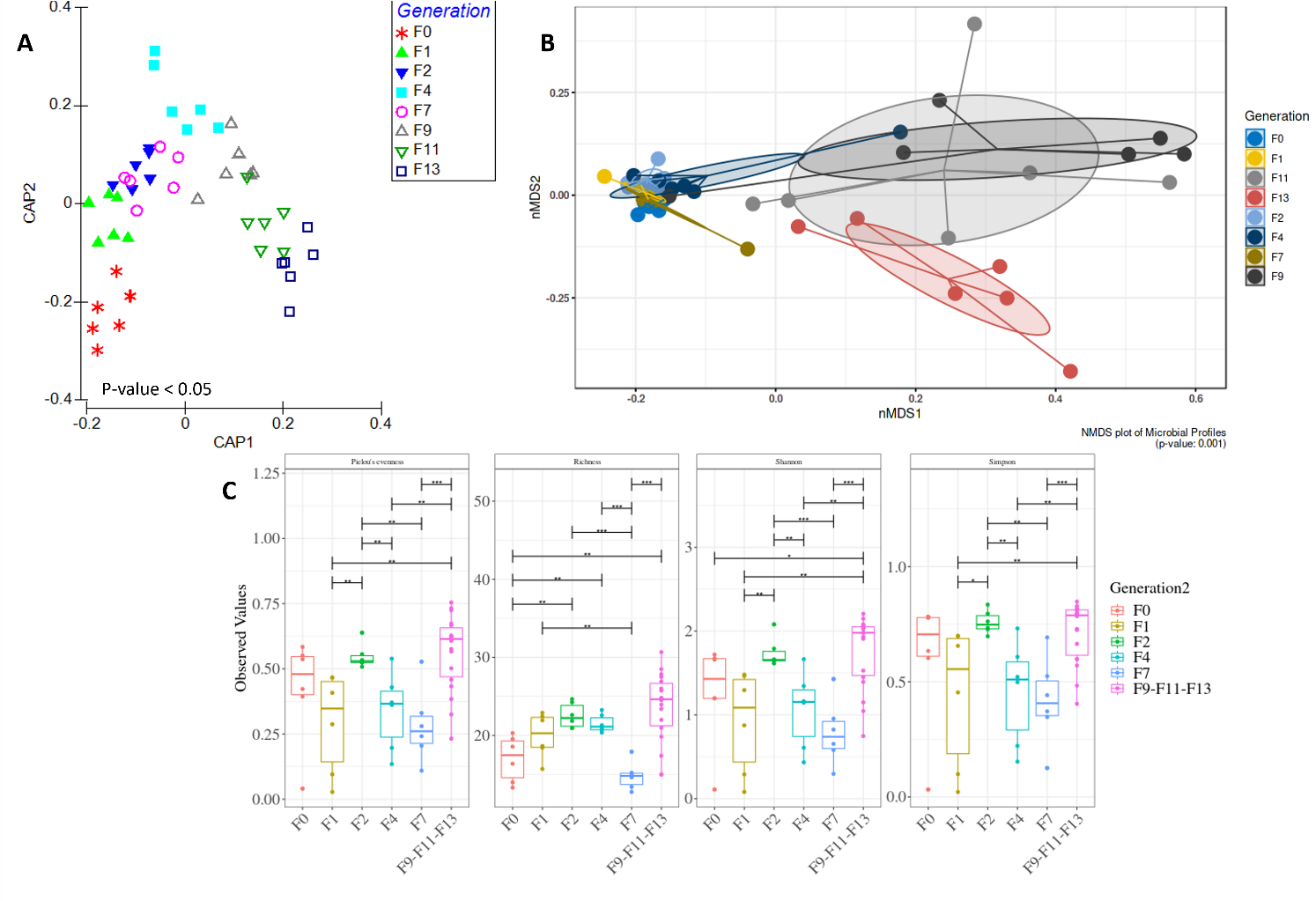


**Supplementary Figure 9:** Diversity of bacterial communities derived from guts of *C. Capitata* teneral during laboratory adaptation of the wild population. CAP **(A)** and NMDS **(B)** analyses were used to find axes that best discriminate the groups of interest. **(C)** Species richness and diversity indices with significance differences, boxes represent the interquartile range (IQR), the line within the boxes is the median, and the dots represent samples.


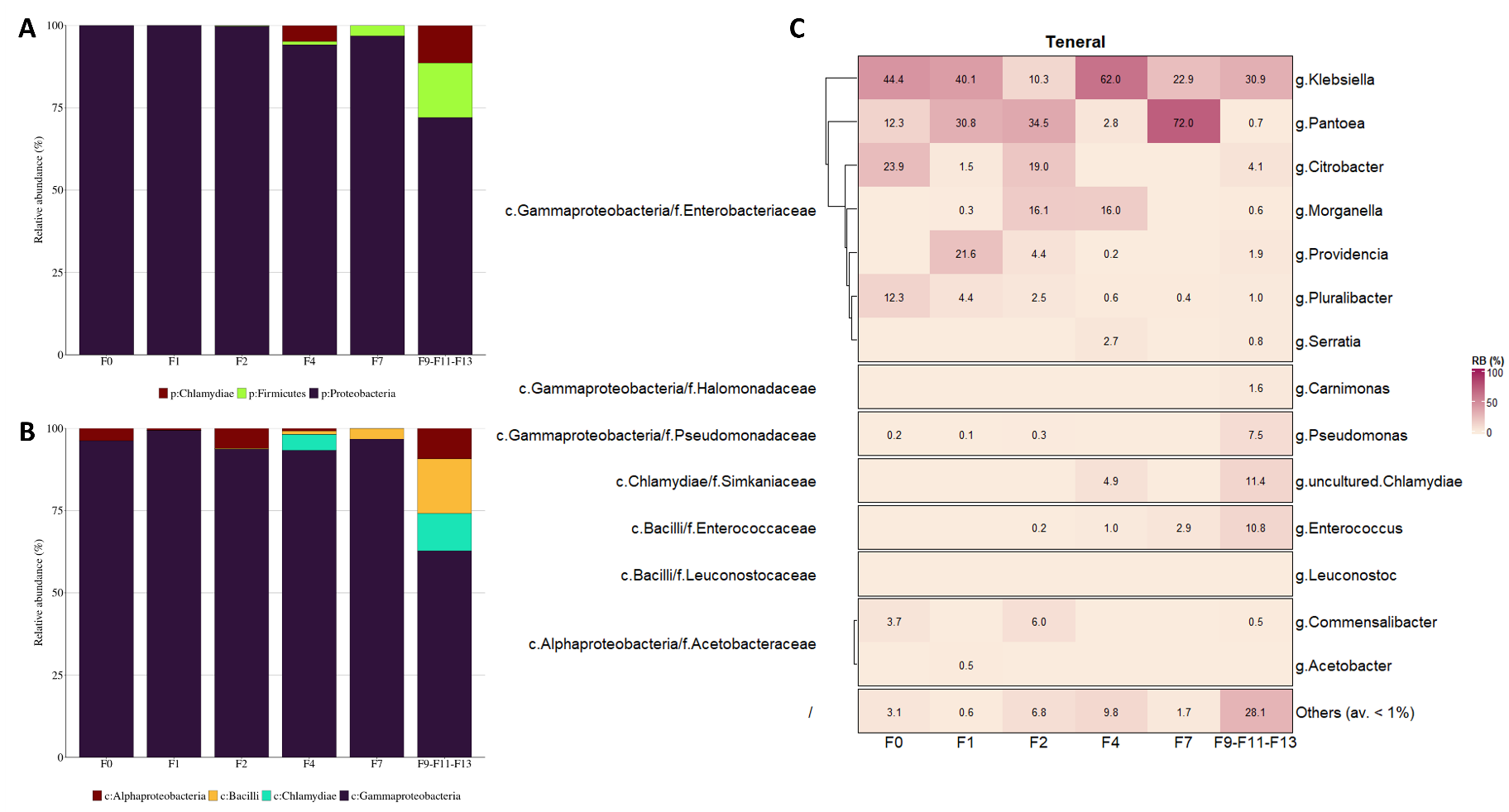


**Supplementary Figure 10:** Composition of bacterial community associated to guts of *C. Capitata* teneral during laboratory adaptation of wild population. **(A)** Phylum level, **(B)** Class level and **(C)** Genus level.


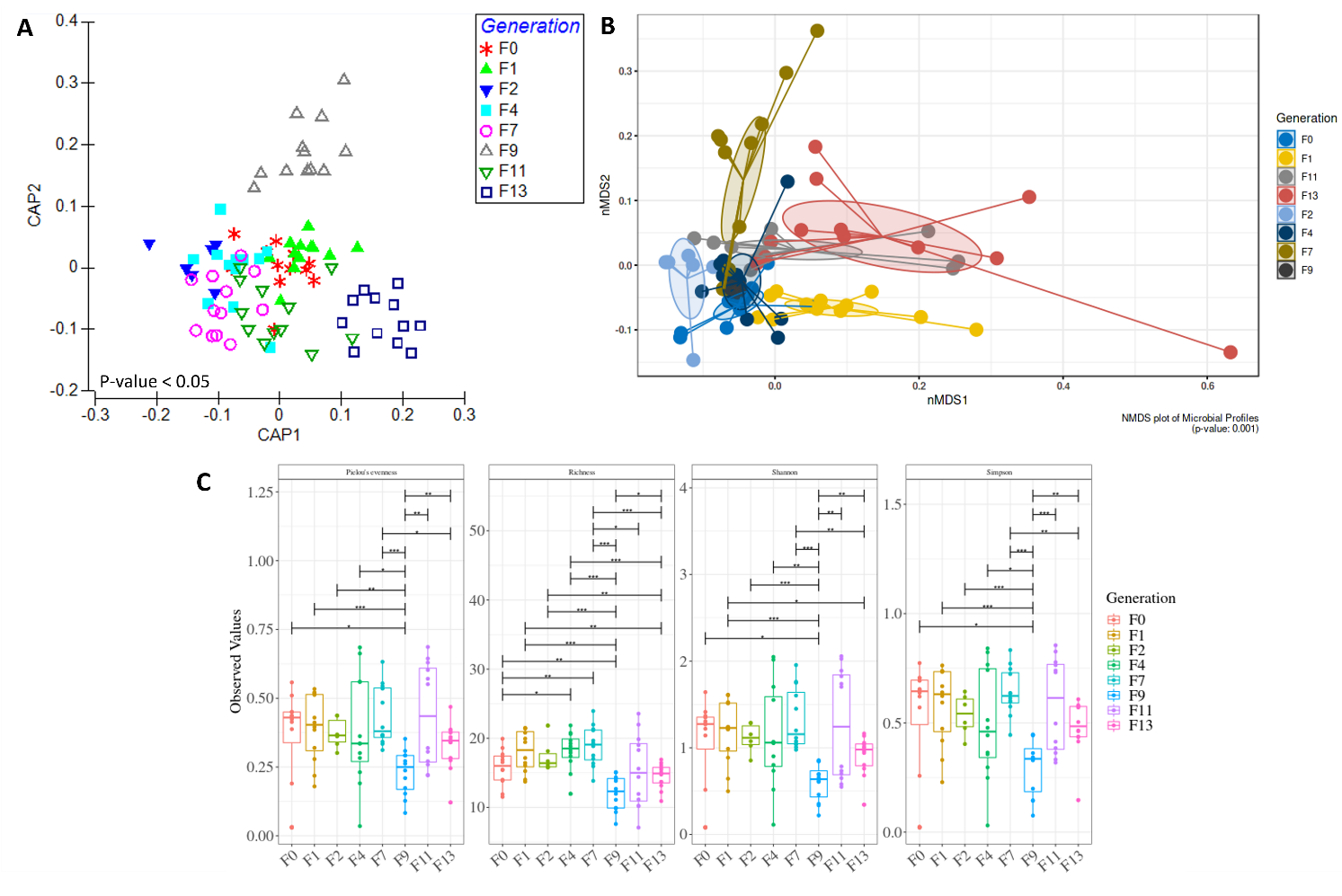


**Supplementary Figure 11:** Diversity of bacterial communities derived from guts of *C. Capitata* adult during laboratory adaptation of wild population. CAP **(A)** and NMDS **(B)** analyses were used to find axes that best discriminate the groups of interest. **(C)** Species richness and diversity indices with significance differences, boxes represent the interquartile range (IQR), the line within the boxes is the median, and the dots represent samples.


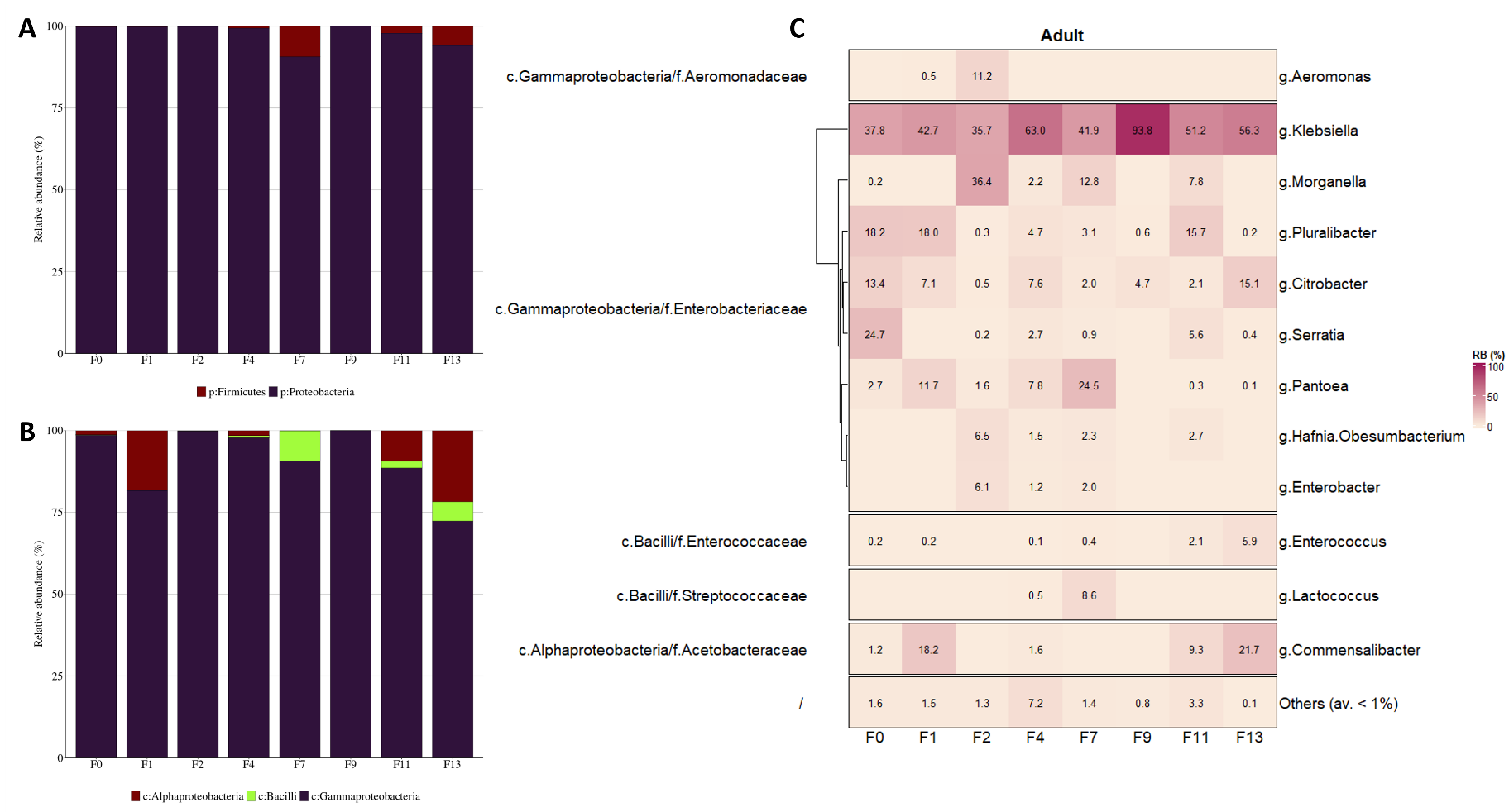


**Supplementary Figure 12:** Composition of bacterial community associated to guts of *C. Capitata* adult during laboratory adaptation of wild population. **(A)** Phylum level, **(B)** Class level and **(C)** Genus level.


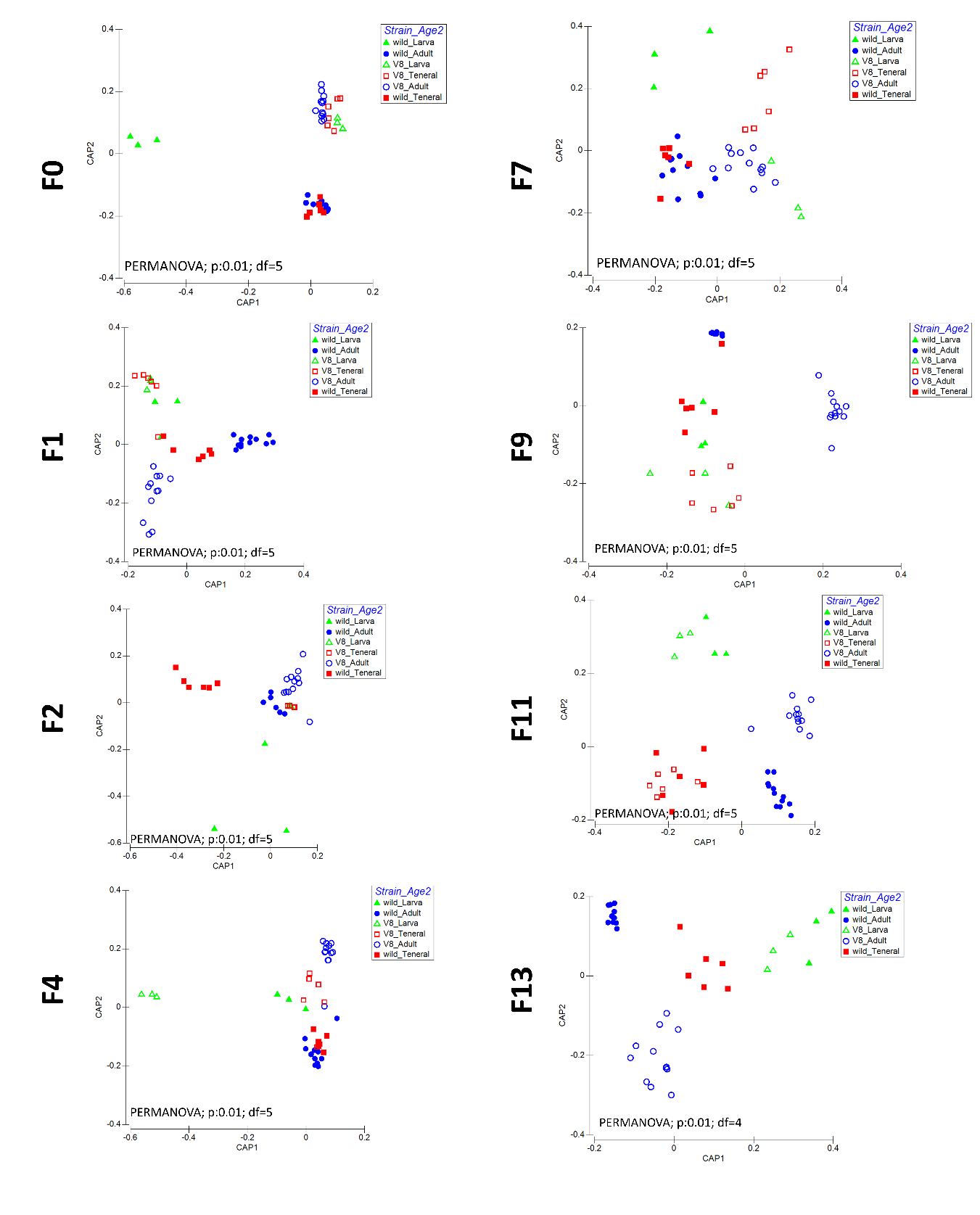


**Supplementary Figure 13:** Changes in bacterial community structure associated to guts of larva, teneral and adults originated from Vienna8FD-GSS (V8) and wild *C. capitata* stains during laboratory adaptation. CAP analysis and PERMANOVA test were performed based on GUniFrac dissimilarity


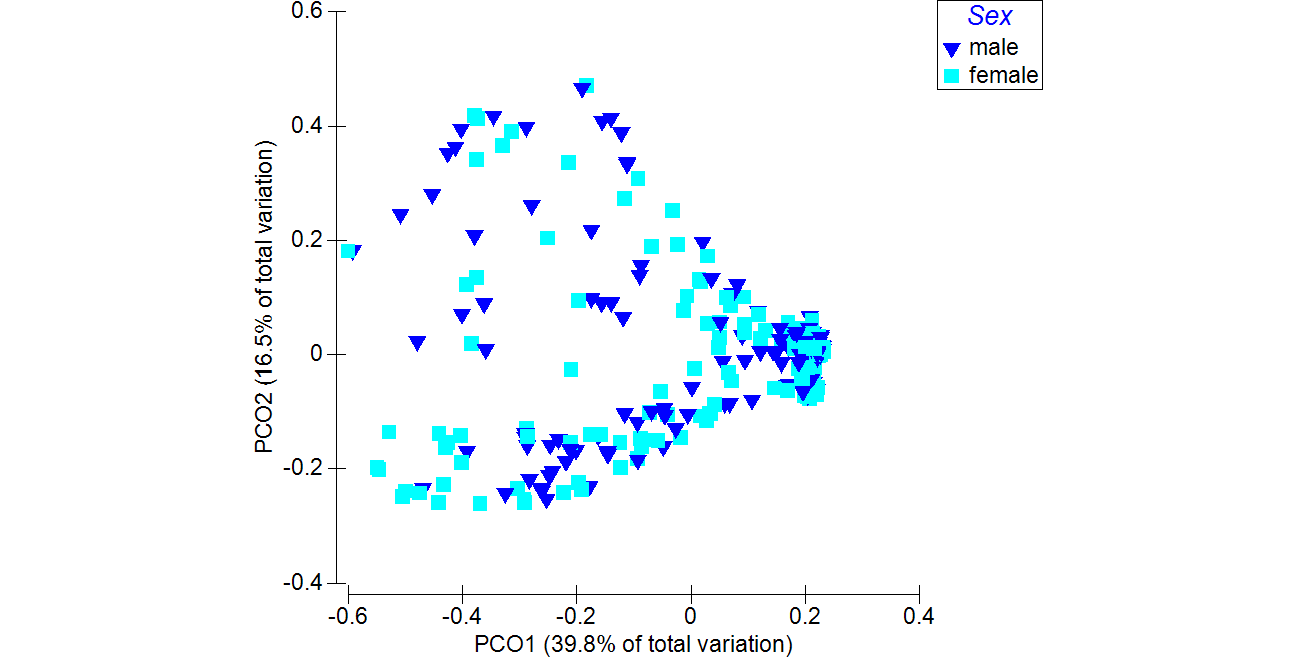
**Supplementary Figure 14:** PCoA representation illustrate the changes in bacterial community structure between males and females of *C. capitata*.
